# Supplementary material for: Structural basis of transmembrane coupling of the HIV-1 envelope glycoprotein
Source: Nat Commun. 2020 May 8;11:2317. doi: 10.1038/s41467-020-16165-0 (PMC7210310; doi:10.1038/s41467-020-16165-0)
Supplement: Supplementary file 1 — Supplementary Information [file 41467_2020_16165_MOESM1_ESM.pdf]

Supplementary Information

Structural basis of transmembrane coupling of the HIV-1 envelope glycoprotein

Alessandro Piai, Qingshan Fu, Yongfei Cai et al.

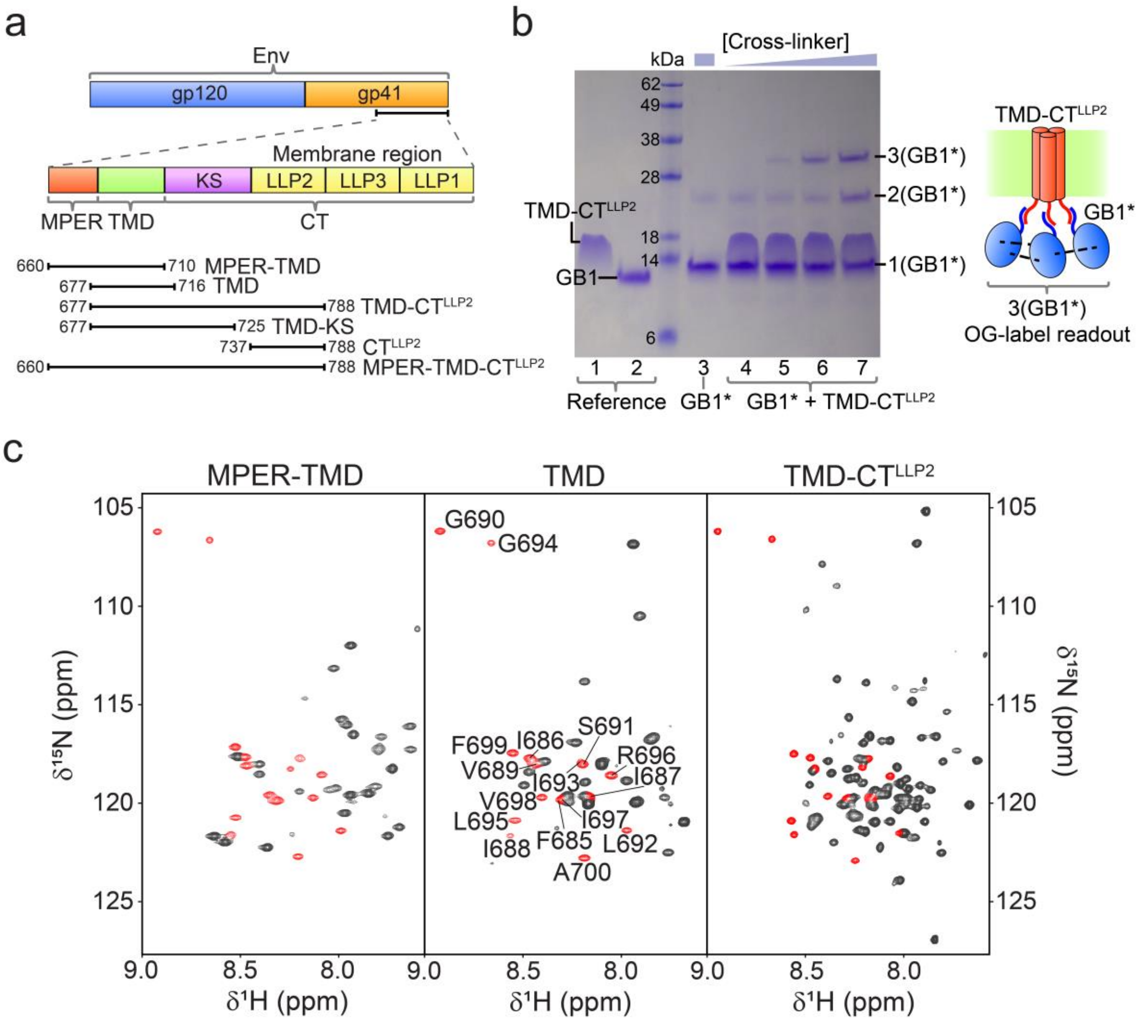

Supplementary Fig. 1 | Biochemical and NMR characterization of TMD-CT^{LLP2} in bicelles. **a,** Designation of the membrane-related components of HIV-1 gp41 (top) and definition of the constructs used in this study (bottom). **b,** Determination of the oligomeric state of TMD-CT^{LLP2} reconstituted in DMPC-DHPC bicelles (q = 0.5) using the OG-label method[1] that uses the GB1 protein as reporter of TMD-CT^{LLP2} oligomerization. The SDS-PAGE gel lanes are: 1 – TMD-CT^{LLP2} (monomeric in SDS); 2 – GB1 (n.b. GB1* denotes GB1 conjugated to a triNTA molecule); 3 – GB1* cross-linked alone; 4-7 – GB1* cross-linked in the presence of TMD-CT^{LLP2} at increasing cross-linker concentration. The experiment was performed once. **c,** Comparison of the 2D ^{1}H-^{15}N TROSY-HSQC spectra of the MPER-TMD, TMD and TMD-CT^{LLP2}, showing strong agreement among the three constructs of the chemical shift of residues 685-700 (red). Source data are provided as a Source Data file.

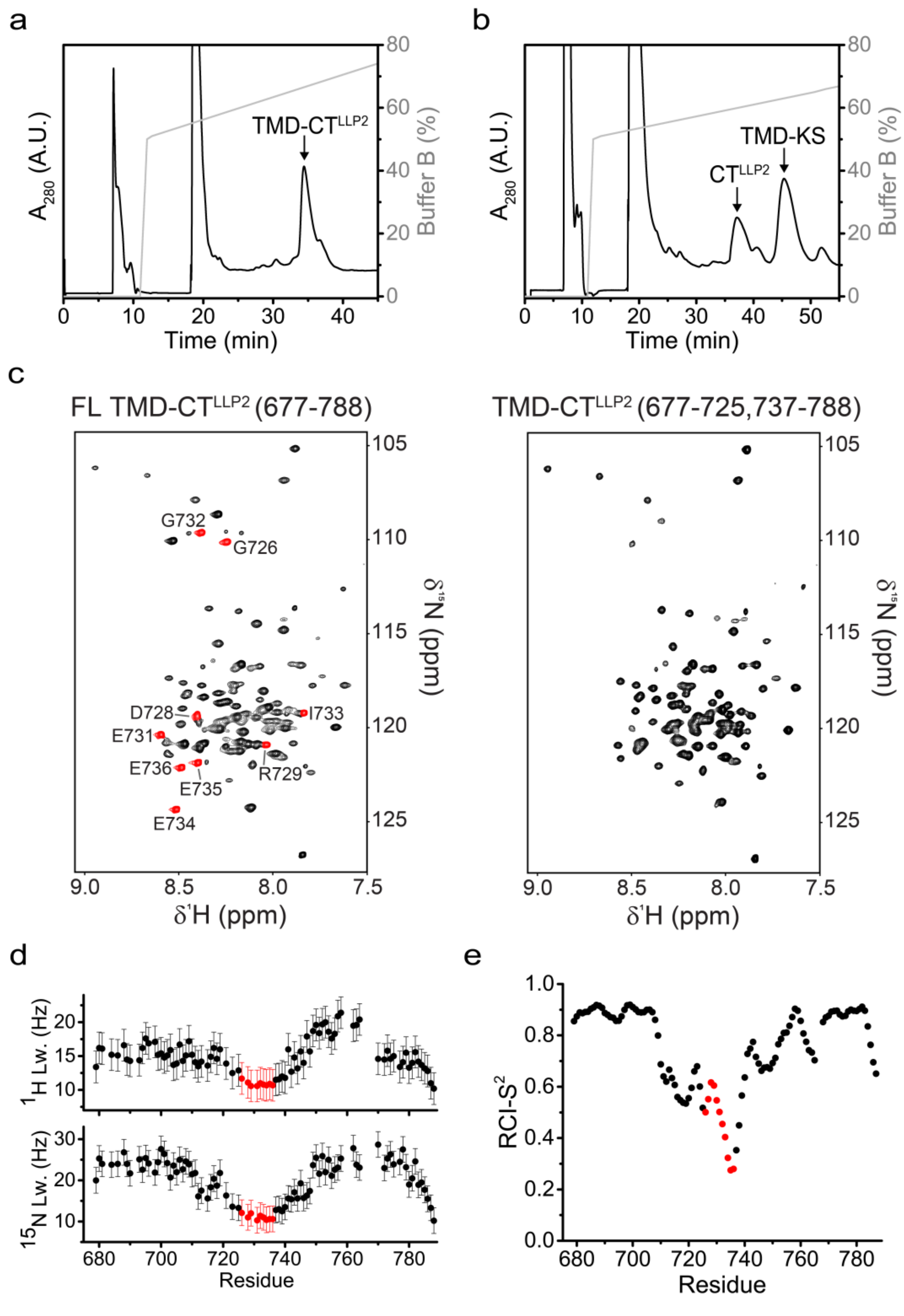

Supplementary Fig. 2 | TMD-CT^{LLP2}, TMD-KS and CT^{LLP2} purification and deletion of residues 726-736. a, RP-HPLC chromatogram of the TMD-CT^{LLP2}. The protein absorbance was measured at 280 and 214 nm (the latter not shown for clarity). The gray line indicates the gradient of the mobile phase composition (% of Buffer B). **b,** Same as (a) but for the TMD-KS and CT^{LLP2}.

The constructs were obtained by introducing in the TMD-CT^{LLP2} an additional Met between the KS and the CT^{LLP}, so that the two fragments were cleaved by CNBr together with the TrpLE. **c,** 2D ^{1}H-^{15}N TROSY-HSQC spectra of the full-length (FL) TMD-CT^{LLP2} (left) and the TMD-CT^{LLP2} with residues 736-736 deleted (right). The cross-peaks of residues 726-736 are shown in red. The two spectra are virtually identical indicating that the deletion did not alter the structure of the protein. **d,** Residue-specific backbone $^{1}H^{N}$ (top) and ^{15}N (bottom) NMR peak linewidth of the FL TMD-CT^{LLP2} obtained from the 2D ^{1}H-^{15}N TROSY-HSQC spectrum in panel (c). Error bars represent the uncertainty derived from cross-peaks signal to noise. The linewidths of residues 726-736, highlighted in red, are significantly smaller than those of the rest of the protein, indicating that the deleted region is highly flexible. **e,** Random coil index (RCI)-based order parameter (S^2) calculated from experimental chemical shifts (^{15}N, $^{13}C^{\alpha}$, $^{13}C^{\beta}$ and $^{13}C'$) using the *MICS* program[2]. The low values computed for residues 726-736 (red dots) indicates the absence of secondary structure in the deleted region. Source data are provided as a Source Data file.

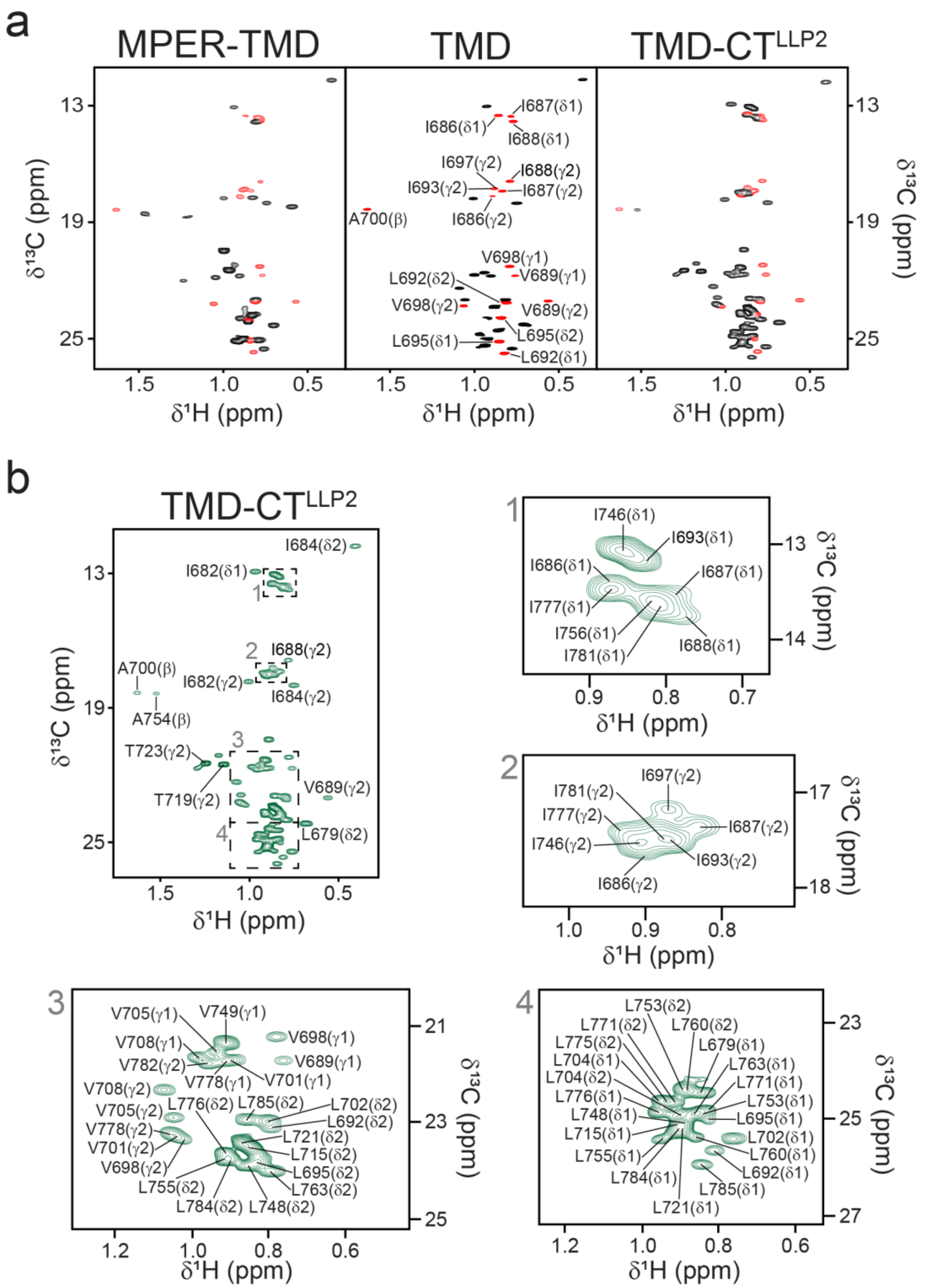

Supplementary Fig. 3 | Methyl group resonances of the MPER-TMD, TMD and TMD-CT^{LLP2} in bicelles. a, Comparison of the methyl group regions of the 2D ^{1}H-^{13}C HSQC spectra of the MPER-TMD, TMD and TMD-CT^{LLP2}, showing strong agreement among the three constructs of the chemical shift of residues 685-700 (red). **b,** Assigned methyl group resonances of the TMD-CT^{LLP2}. The assignment of the most crowded regions is shown in the four expansions.

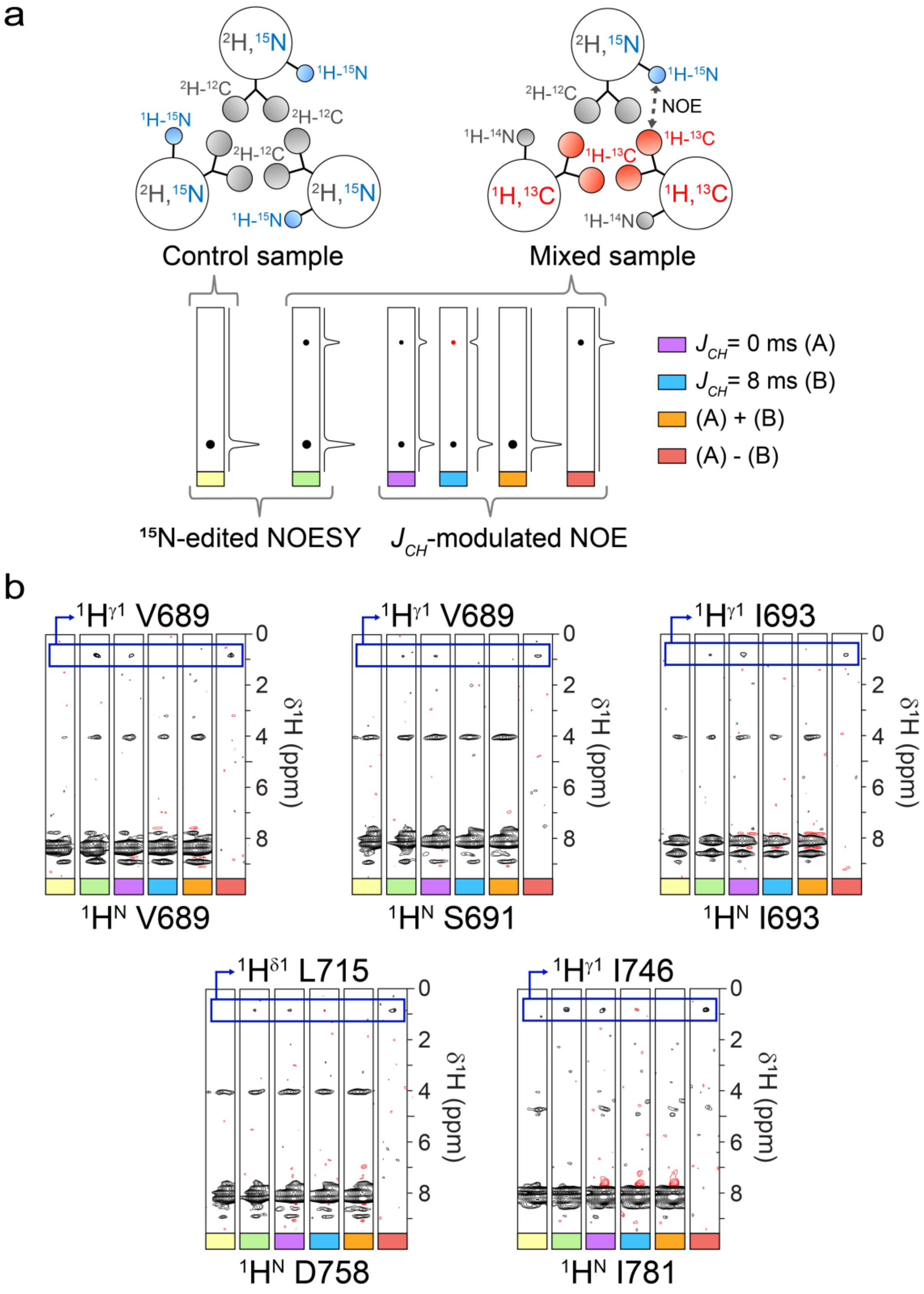

Supplementary Fig. 4 | Identification of unambiguous inter-chain NOEs of the TMD-CT^{LLP2}. **a,** Schematic representation of the samples used to record the NOESY experiments. The control sample (left) contained only ^{15}N, ^{2}H-labeled TMD-CT^{LLP2}, and the mixed sample (right) contained ~1:1 ratio of ^{15}N, ^{2}H-labeled TMD-CT^{LLP2} to ^{13}C-labeled TMD-CT^{LLP2}. ^{1}H-^{15}N and ^{1}H-^{13}C groups

are shown in blue and red, respectively, whereas the undetectable groups (e.g., ^{1}H-^{14}N and ^{2}H-^{12}C) are shown in gray. The mixed sample was used to detect inter-chain NOEs from ^{1}H-^{15}N to ^{1}H-^{13}C. In the bottom, the expected diagonal and cross-peaks in each experiment are schematically illustrated, with positive and negative peaks in black and red, respectively. **b,** NOESY strips showing examples of the unambiguous inter-chain NOEs of the TMD-CT^{LLP2}, identified using the above strategy. The control sample was used to acquire a 3D ^{15}N-edited NOESY-TROSY-HSQC spectrum, while the mixed sample was used to record an identical ^{15}N-edited NOESY-TROSY-HSQC spectrum as well as the 3D J_{CH}-modulated NOE spectrum. A mixing time of 200 ms was employed in all the experiments. Comparison of the strips from the ^{15}N-edited NOESY-TROSY-HSQC spectra of the control and mixed samples allowed the identification of inter-chain NOEs. The identified NOEs were then unambiguously validated by recording the J_{CH}-modulated NOE spectrum, in which two interleaved spectra were acquired with varying the J_{CH} evolution (J_{CH} = 0 ms and J_{CH} = 8 ms) before the NOE mixing. The subtraction of the two spectra allowed selection of only the inter-protomer NOEs. Positive and negative peaks are shown in black and red, respectively. The inter-protomer NOEs shown are: $^{1}H^{N}$ V689 to $^{1}H^{\gamma1}$ V689; $^{1}H^{N}$ S691 to $^{1}H^{\gamma1}$ V689; $^{1}H^{N}$ I693 to $^{1}H^{\gamma1}$ I693; $^{1}H^{N}$ D758 to $^{1}H^{\delta1}$ L715; $^{1}H^{N}$ I781 to $^{1}H^{\gamma1}$ I746.

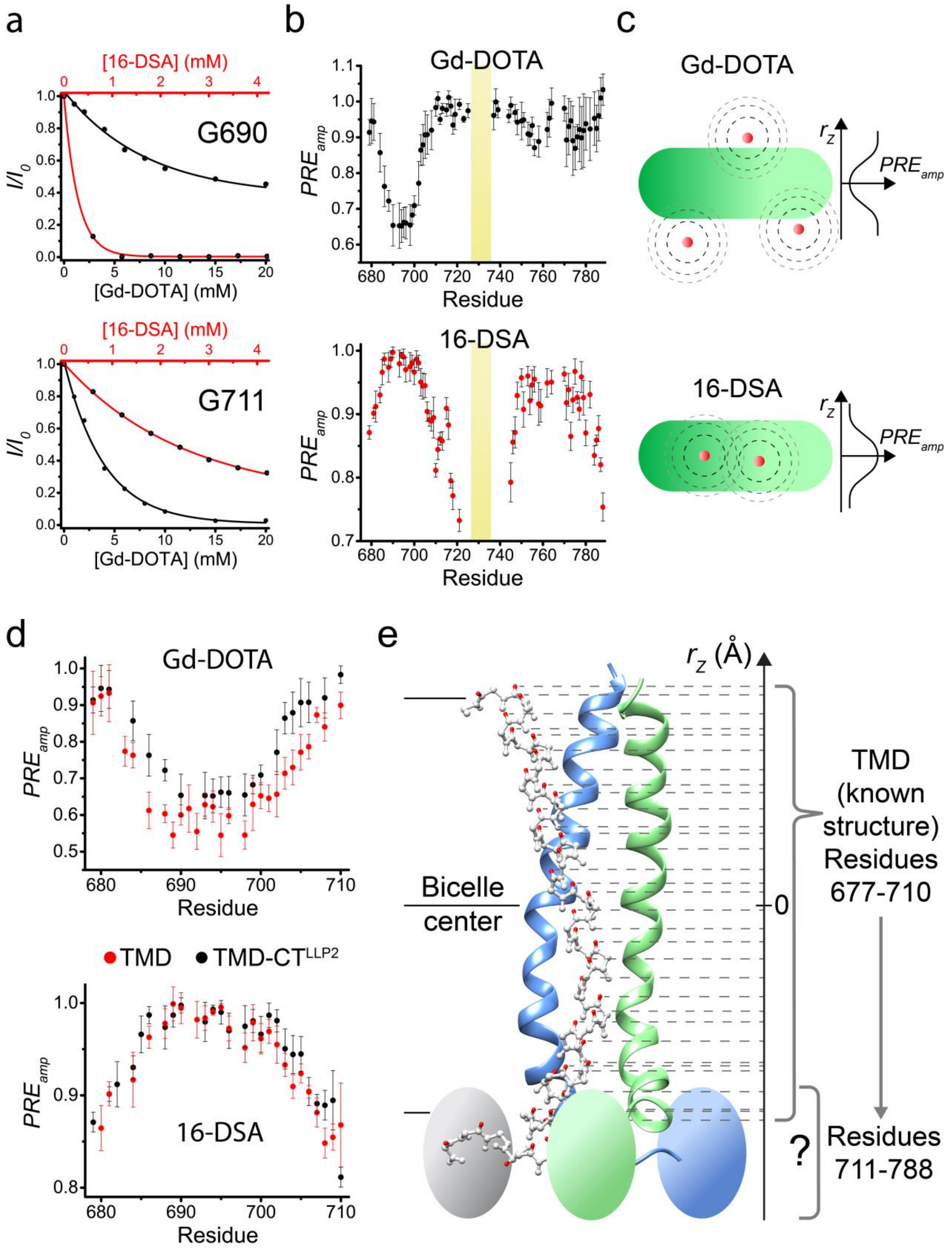

Supplementary Fig. 5 (continue in the next page)

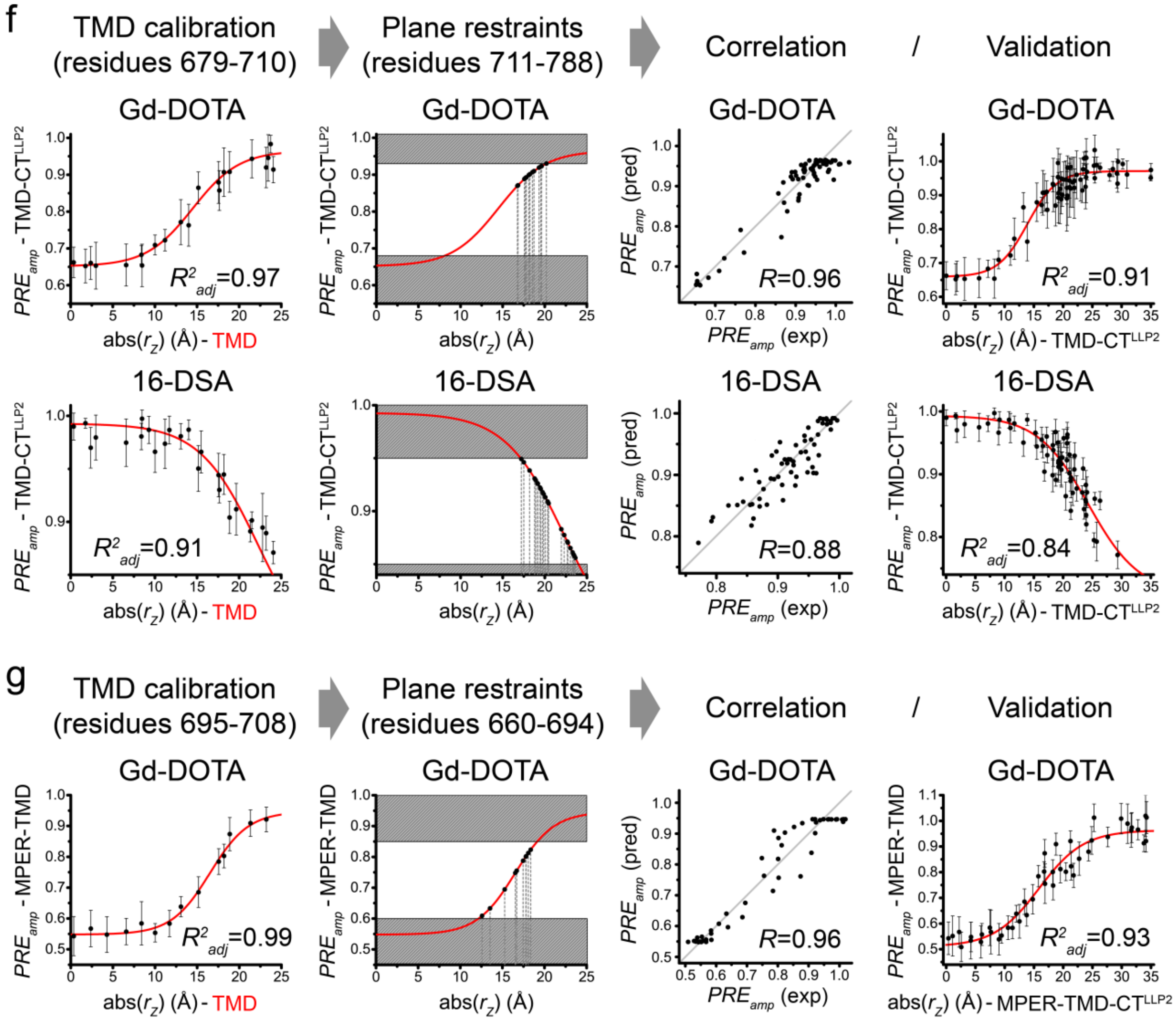

Supplementary Fig. 5 | Derivation of the plane restraints used in the TMD-CT^{LLP2} and MPER-TMD-CT^{LLP2} structure calculation. a, The I/I_0 vs. [Gd-DOTA] (black) and I/I_0 vs. [16-DSA] (red) plots for residues G690 (buried) and G711 (exposed) of the TMD-CT^{LLP2} are shown as examples. Data fitting (black and red lines for Gd-DOTA and 16-DSA, respectively) to the exponential decay function (Eq. 1) yielded the PRE_{amp} of the two residues. Variation in signal decays reflects the different localization of the two residues. **b,** PRE_{amp} vs (residue number) plots for TMD-CT^{LLP2} obtained from Gd-DOTA (black, top) and 16-DSA (red, bottom) titrations. Error bars represent the uncertainty derived from fitting error. Missing values are due to prolines or residues with overlapping peaks. The yellow box marks the deleted residues 726-736. **c,** Schematic illustration of the distribution of Gd-DOTA (top) and 16-DSA (bottom) relative to the bicelle, with the expected reciprocal PRE_{amp} profiles shown on the right. **d,** Comparison of the PRE_{amp} vs (residue number) plots of residues 679-710 between the TMD-CT^{LLP2} (black) and the TMD (red), derived from Gd-DOTA (top) and 16-DSA (bottom) titrations. Error bars represent the uncertainty derived from fitting error. The two data sets collectively indicate that residues 679-710 have the same membrane partition in both constructs. **e,** Schematic illustration of the strategy used to assign plane restraints for residues 711-788 of the TMD-CT^{LLP2}. The close agreement in the TM region

between the TMD-CT^{LLP2} and TMD constructs allowed to calibrate the PRE_{amp} values of the TMD-CT^{LLP2} against the previously established structure and membrane partition of the TMD[3,4] such that the PRE_{amp} values can directly translate to plane restraints for the CT region of the TMD-CT^{LLP2}. **f,** Assignment of plane restraints for residues 711-788 of the TMD-CT^{LLP2} using data from Gd-DOTA (top) and 16-DSA (bottom) titrations. First, the PRE_{amp} vs. *residue number* of the TMD-CT^{LLP2} was converted to PRE_{amp} vs. r_Z using the known TMD trimer structure (PDB ID: 5JYN). The PRE_{amp} vs. r_Z profiles were then fitted to the sigmoidal function (Eq. 2) (left). Second, the best-fit curves (red) were used to assign r_Z for residues 711-788 (middle). Only r_Z in the PRE-sensitive regions of the curves were retained (white area). Finally, the calculated r_Z from Gd-DOTA and 16-DSA curves were averaged, merged into one single data set and used as plane restraints for the TMD-CT^{LLP2} structure calculation. Once the structure was obtained, the correlation between experimental PRE_{amp} and those predicted from the new structure was determined. For both Gd-DOTA and 16-DSA data sets, the agreement is good as high Pearson linear correlation coefficient (R) were obtained (note: R is smaller for 16-DSA than for Gd-DOTA because the magnitude of PRE_{amp} variation is smaller for the former (~0.8 - 1.0) than for the latter (~0.6 - 1.0)). In addition, the membrane partition of the TMD-CT^{LLP2} trimer was derived by fitting the PRE_{amp} vs. r_Z profile (generated this time using the new atomic coordinates) to Eq. 2 and found to be virtually identical to that of the TMD, validating the use of the latter as internal calibration. In all plots, error bars represent the uncertainty derived from fitting error. **g,** Same as (f) but for the MPER-TMD-CT^{LLP2}. In this case, only the Gd-DOTA data were used. Source data are provided as a Source Data file.

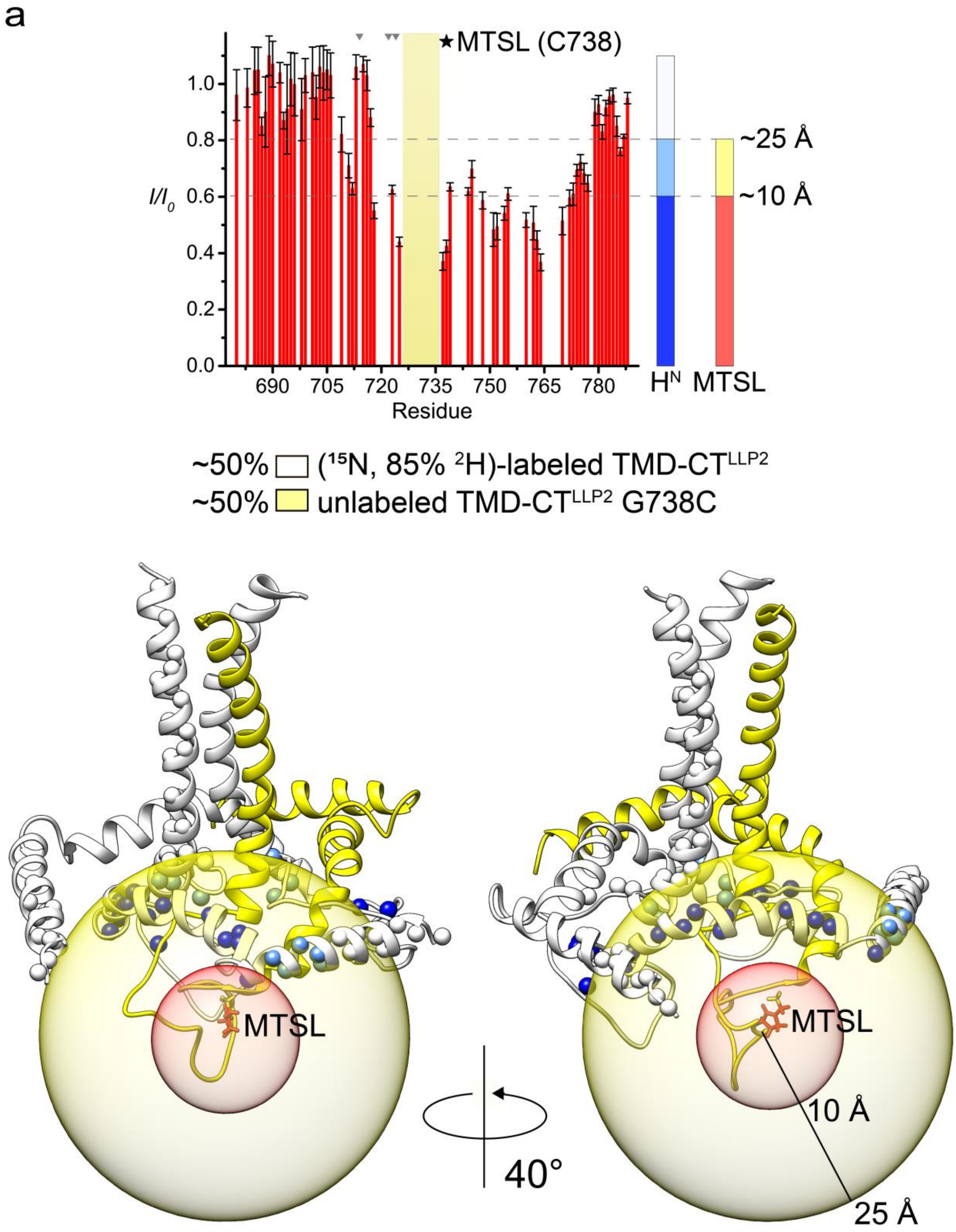

Supplementary Fig. 6 (continue in the next page)

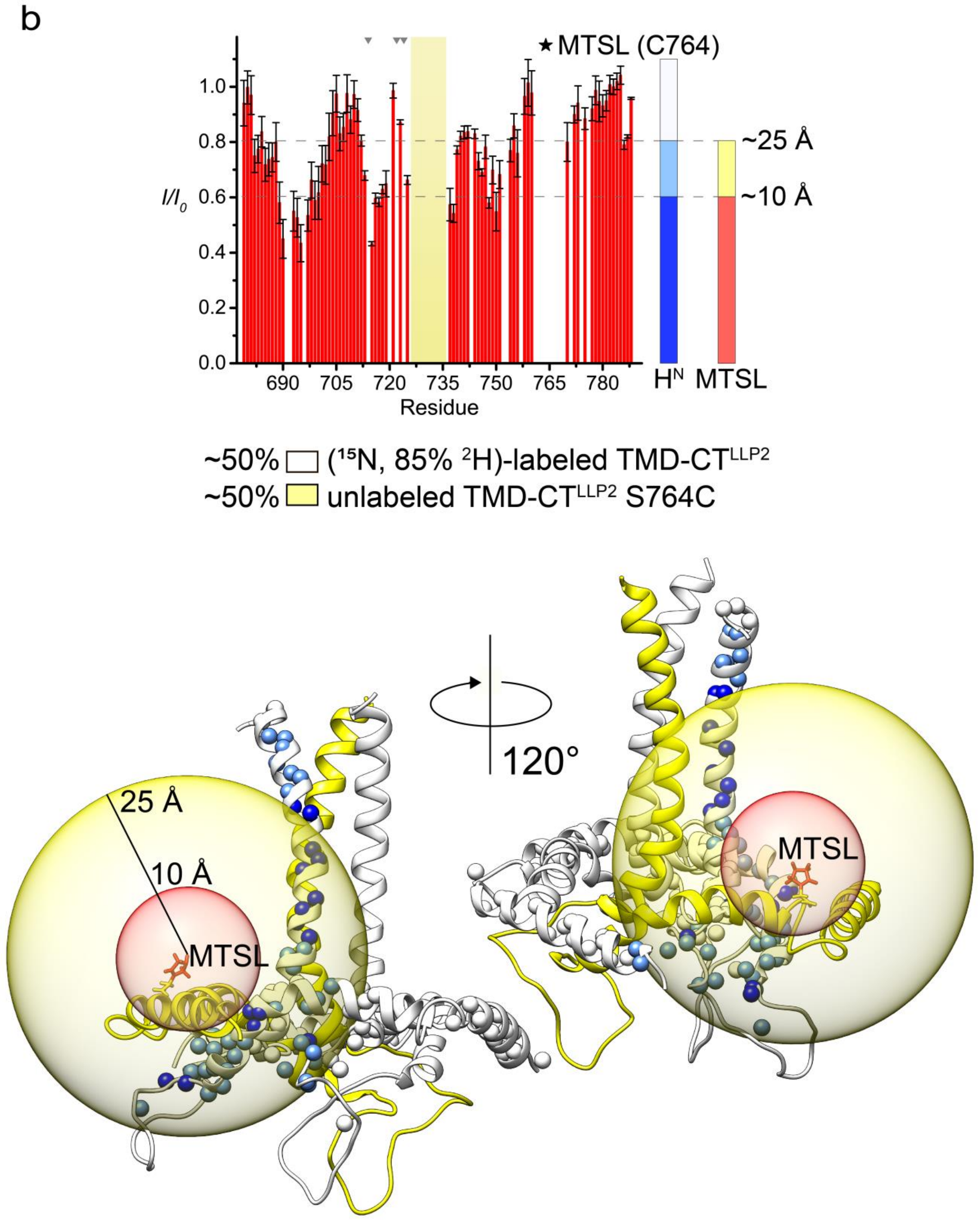

Supplementary Fig. 6 (continue in the next page)

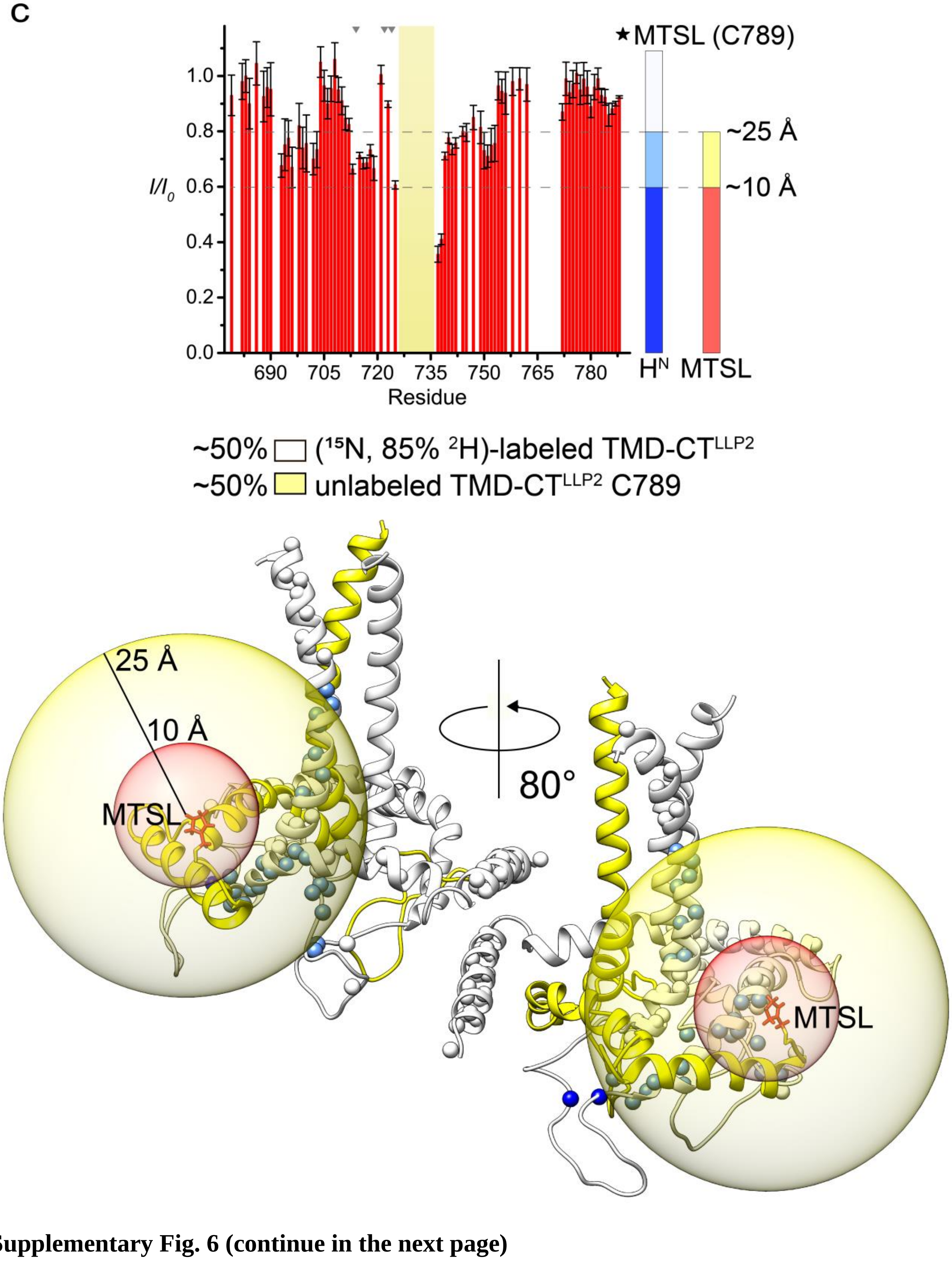

Supplementary Fig. 6 (continue in the next page)

d

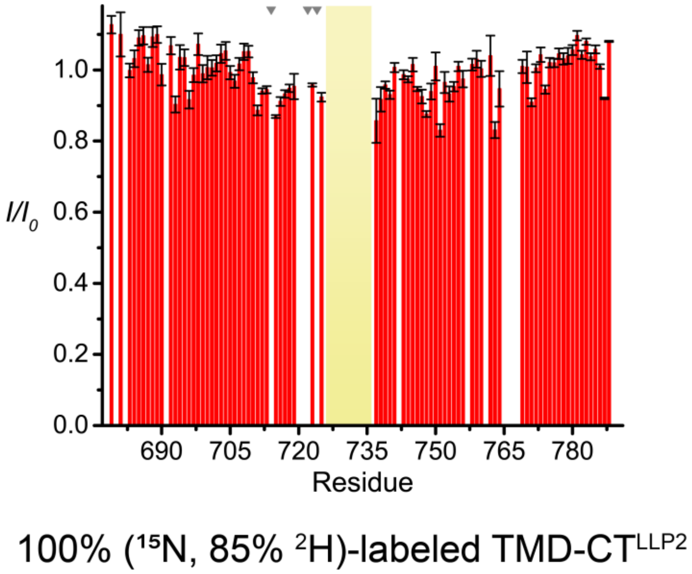

Supplementary Fig. 6 | Validation of the TMD-CT^{LLP2} structure by inter-chain PRE analysis. a, *Top*: Residue-specific PRE (I/I_0) plot of (^{15}N, 85% ^{2}H)-labeled TMD-CT^{LLP2} mixed with the TMD-CT^{LLP2} G738C mutant MTSL-labeled at C738 at ~1:1 molar ratio. Error bars represent the uncertainty derived from cross-peaks signal to noise. Missing bars are due to prolines or residues with overlapping peaks. The yellow box corresponds to residues 726-736 (deleted in the constructs), the grey triangles mark the proline residues and the asterisk indicates the position of the MTSL label. The horizontal dash lines define the three PRE regimes used to map onto the protein structure (bottom): strong ($I/I_0 \leq 0.6$; blue), medium ($0.6 < I/I_0 \leq 0.8$; light blue) and weak ($I/I_0 > 0.8$; white). PRE of 0.6 and 0.8 correspond approximately to a distance of 10 and 25 Å, respectively, from the paramagnetic tag. *Bottom*: Mapping of the PRE regimes onto the protein structure using the color scheme above. The (^{15}N, 85% ^{2}H)-labeled TMD-CT^{LLP2} and the MTSL-labeled G738C mutant are represented as white and yellow ribbons, respectively. Amide protons for which accurate PRE measurement was feasible are shown as spheres and colored according to the PRE regimes defined above. The yellow and red spheres cover 25 and 10 Å distance from the paramagnetic tag (highlighted in red), respectively. **b,** Same as (a) but for MTSL labeled at C764. **c,** Same as (a) but for MTSL labeled at C789. **d,** Control experiment in which only (^{15}N, 85% ^{2}H)-labeled TMD-CT^{LLP2} was used. The absence of PRE effect indicates that the sample preparation procedure completely removed excessive free MTSL (note: this is important as MTSL is hydrophobic and partitions in micelles or bicelles). Source data are provided as a Source Data file.

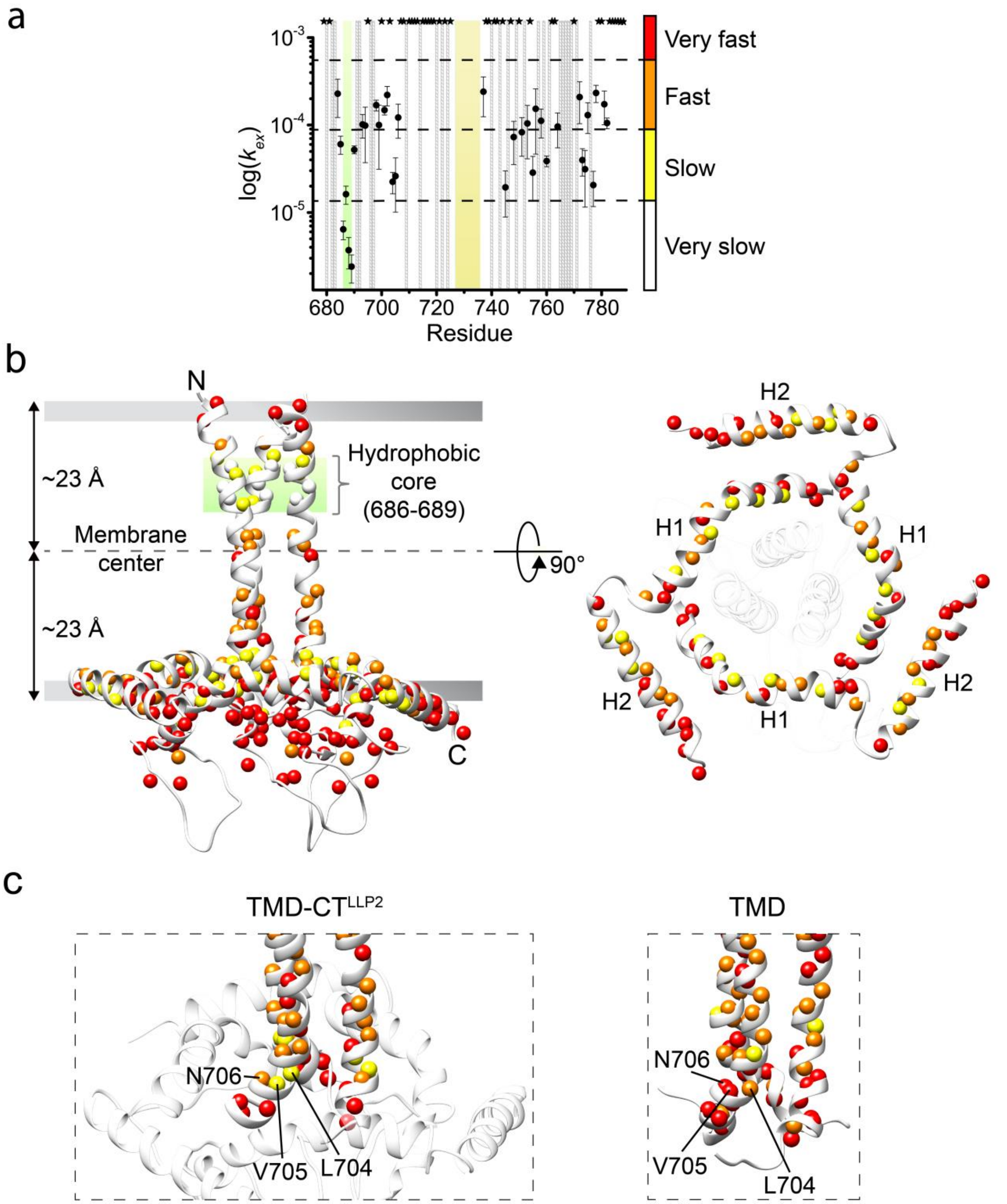

Supplementary Fig. 7 | H-D exchange of the TMD-CT^{LLP2} in *q* = 0.5 bicelles at 303 K. a, Residue-specific k_{ex} reported in logarithmic scale. Error bars represent the uncertainty derived from fitting error. Amide protons with exchange too fast to be measured are marked as ★. Residues that could not be analyzed (overlapping peaks and prolines) are marked with striped gray bars. The yellow box corresponds to deleted residues 726-736. The green box marks the hydrophobic core

(residues 686-689) that exhibits very slow exchange. The color code on the right represents the four different exchange regimes: very fast (red), fast (orange), slow (yellow), and very slow (white: very slow). **b,** Mapping of the four exchange regimes defined in (a) onto the TMD-CT^{LLP2} trimer structure, placed in lipid bilayer according to the PPT-derived membrane partition. The location of the hydrophobic core (residues 686-689) is highlighted with the green box. Slow-exchanging residues are mostly facing the bilayer interior, whereas very fast-exchanging residues are mostly outside the bilayer or within the TMD hydrophilic core[4]. **c,** Comparison of the water accessibility of the TM hydrophilic core between the TMD-CT^{LLP2} (left) and TMD [4] (right). Notably, residues 704-706, rapidly exchanging in the TMD, show significantly slower exchange in the TMD-CT^{LLP2} due to protection by the H1 helix of the CT^{LLP2}.

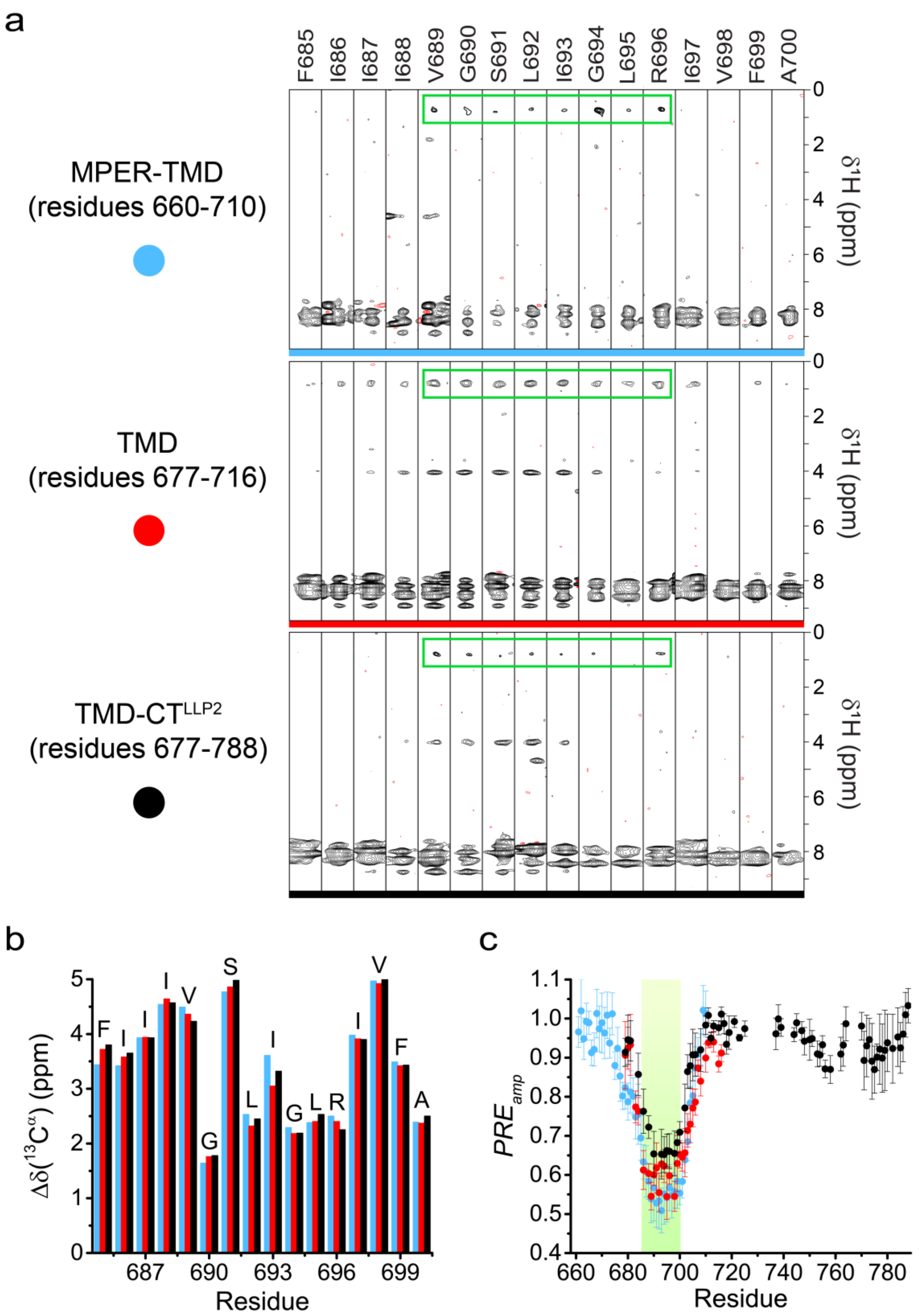

Supplementary Fig. 8 | Structural similarity of the TM core (residues 685-700) among the MPER-TMD, TMD and TMD-CT^{LLP2}. a, The strips corresponding to residues 685-700 taken from the 3D ^{15}N-edited NOESY-TROSY-HSQC spectra of the mixed samples of MPER-TMD (top), TMD (center) and TMD-CT^{LLP2} (bottom), showing similar pattern of inter-chain NOE cross-

peaks. All spectra were recorded with mixed samples containing ~1:1 ratio of (^{2}H, ^{15}N)-labeled and ^{13}C-labeled chains. The same NOE mixing time of 200 ms was employed for all three experiments. The strongest inter-chain NOEs are highlighted in the green box (residues 689-696). The different peak intensities are due to the different size and relaxation properties of the three constructs. The spectra of the MPER-TMD and TMD were taken from previous studies[3,5]. **b,** Comparison of secondary $^{13}C^{\alpha}$ chemical shifts ($\Delta\delta$, experimental chemical shift minus the corresponding "random coil" shift) for residues 685-700 among the MPER-TMD (light blue), TMD (red) and TMD-CT^{LLP2} (black). The remarkable similarity in $\Delta\delta(^{13}C^{\alpha})$ indicates that the three constructs have the same secondary structure in the TM region. **c,** Overlay of the PRE_{amp} vs. *residue number* plots of the three constructs derived from Gd-DOTA titration, showing essentially the same membrane partition profiles for residues 685-700 (green box). The color definition of the three plots is the same as in (b). The notable agreement of the position of the PRE_{amp} minima indicates that the three constructs have virtually identical membrane partition. Error bars represent the uncertainty derived from fitting error. Source data are provided as a Source Data file.

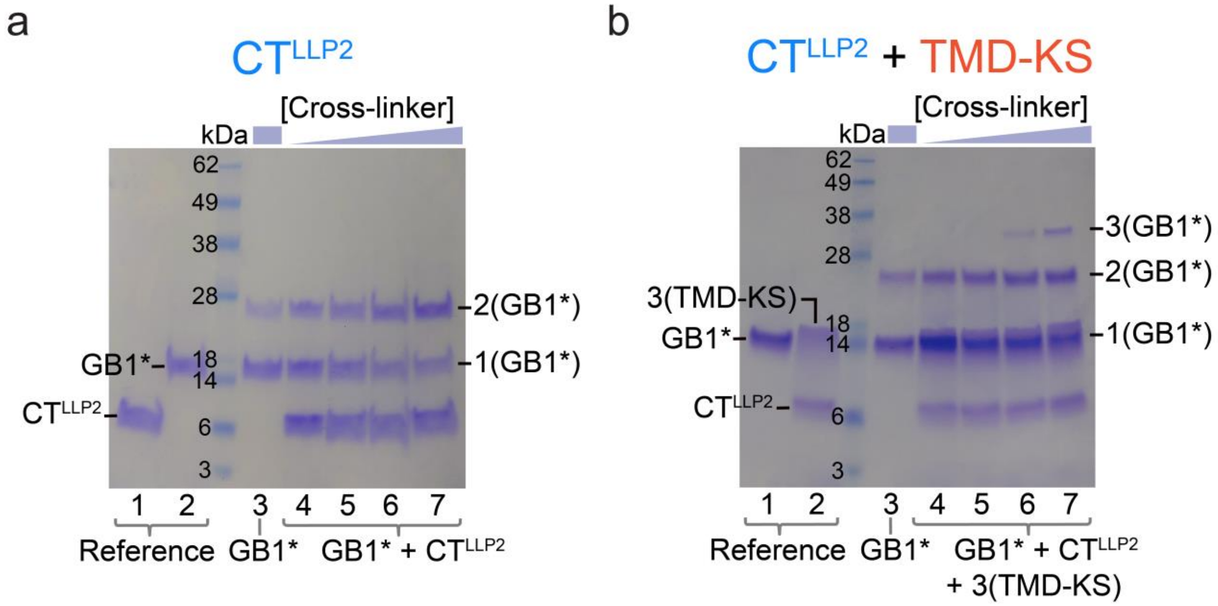

Supplementary Fig. 9 | Evidence of CT^{LLP2}–TMD interaction from OG-label analysis. a, Determination of the oligomerization state of the CT^{LLP2} reconstituted in bicelles by OG-label analysis (see Methods and Supplementary Fig. 1b for OG-label description). The SDS-PAGE gel lanes are: 1 – CT^{LLP2} (monomeric in SDS); 2 – GB1*; 3 – GB1* cross-linked alone; 4-7 – GB1* cross-linked in the presence of CT^{LLP2} at increasing cross-linker concentration. The increasing and decreasing strength of the dimer and monomer bands in lanes 4-7, respectively, compared to those in the control lane 3, suggests that the CT^{LLP2} can form dimers. The experiment was performed once. **b,** Oligomerization state of the CT^{LLP2} reconstituted in bicelles in the presence of the TMD-KS by OG-label analysis. The gel lanes are: 1 – GB1*; 2 – TMD-KS and CT^{LLP2} (trimeric and monomeric is SDS); 3 – GB1* cross-linked alone; 4-7 – GB1* cross-linked in the presence of CT^{LLP2} and TMD-KS at increasing cross-linker concentration. The experiment was performed once. Source data are provided as a Source Data file.

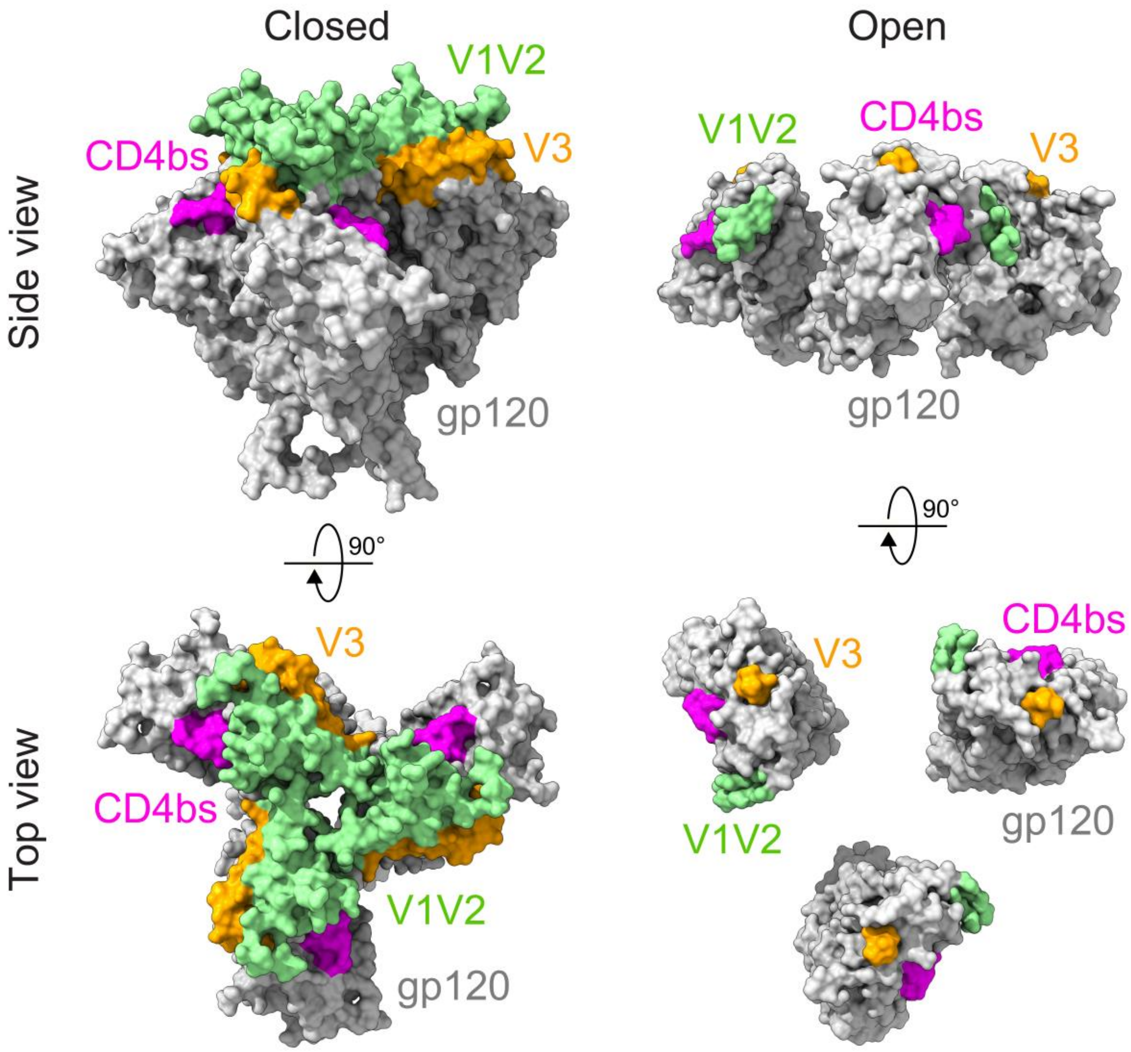

Supplementary Fig. 10 | Epitope mapping of the antibodies used in the pseudovirus-based neutralization assay. Side and top views of gp120 in the closed (prefusion state, left)[6] and open (CD4-bound)[7] conformations are shown. Locations of V1V2 loops, V3 loop and CD4 binding site (CD4bs) are highlighted in green, orange and magenta, respectively. Trimer-specific bnAbs such as PG9, PG16 and PGT145 can bind to the V1V2 loops only when the Env is in the closed conformation, while nonneutralizing or strain-specific neutralizing antibodies such as b6 (CD4bs), 3791 (V3) and 17b (CD4-induced) can bind to the Env only in the open conformation.

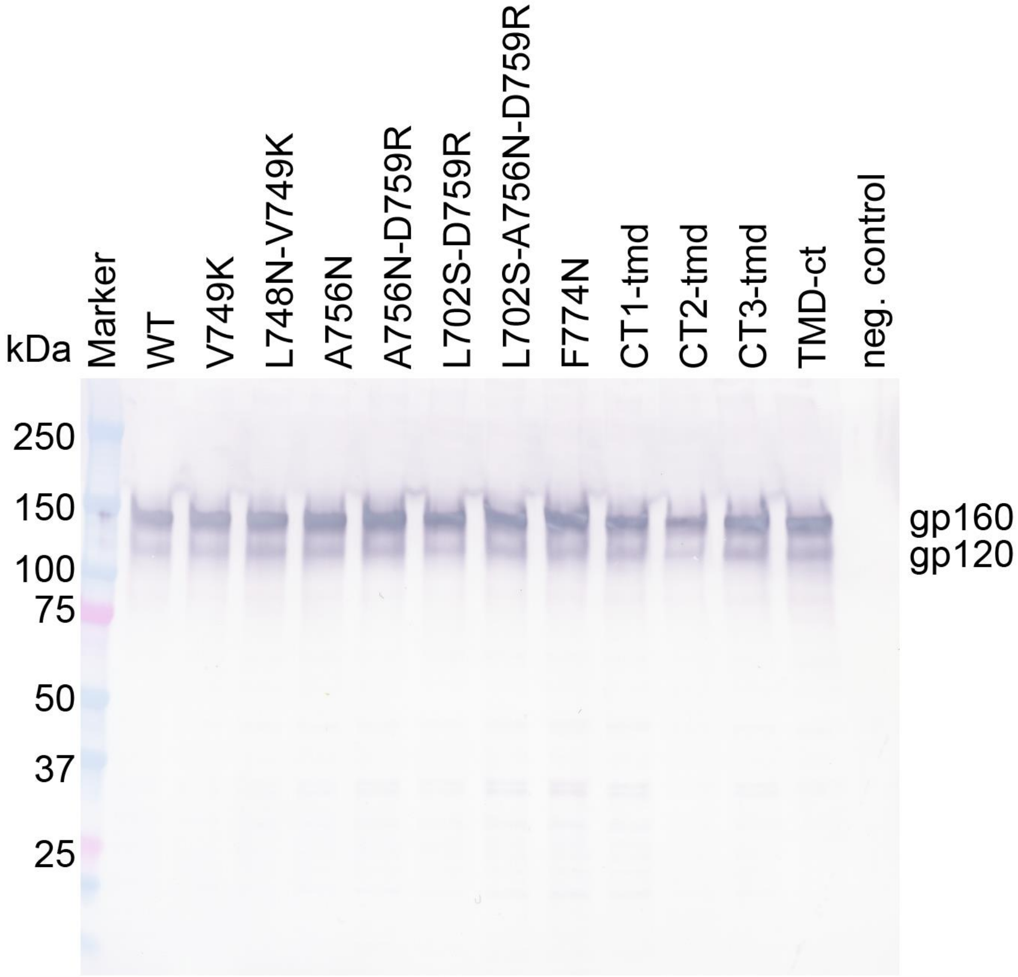

Supplementary Fig. 11 | Expression and processing of Env mutants expressed in 293T cells. Env samples prepared from 293T cells transiently transfected with 1 μg of the HIV-1 gp92UG037.8 gp160 expression plasmid or of each CT mutant were detected by an anti-V3 antibody 3791. The experiment was repeated independently with similar results twice. Source data are provided as a Source Data file.

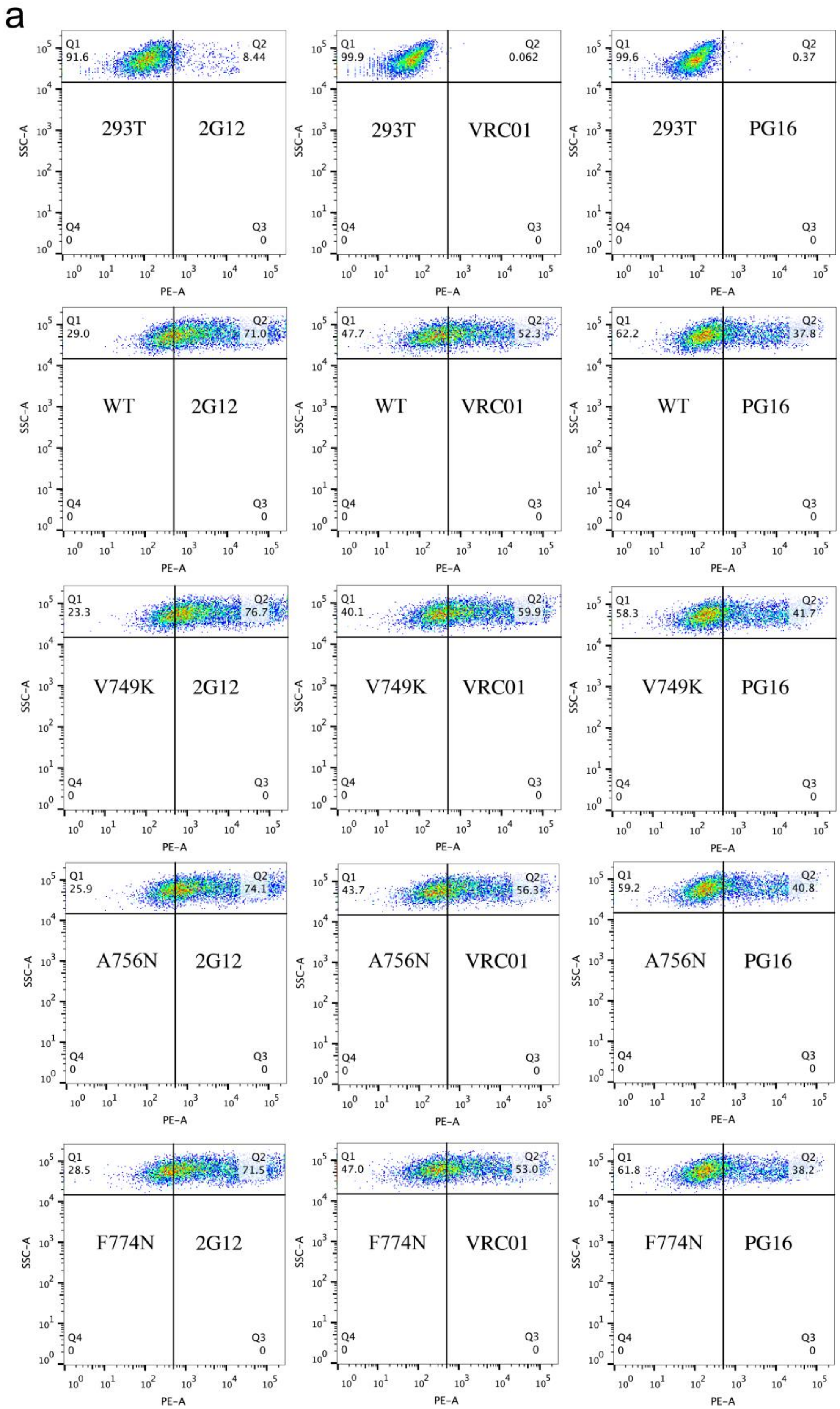

Supplementary Fig. 12 (continue in the next page)

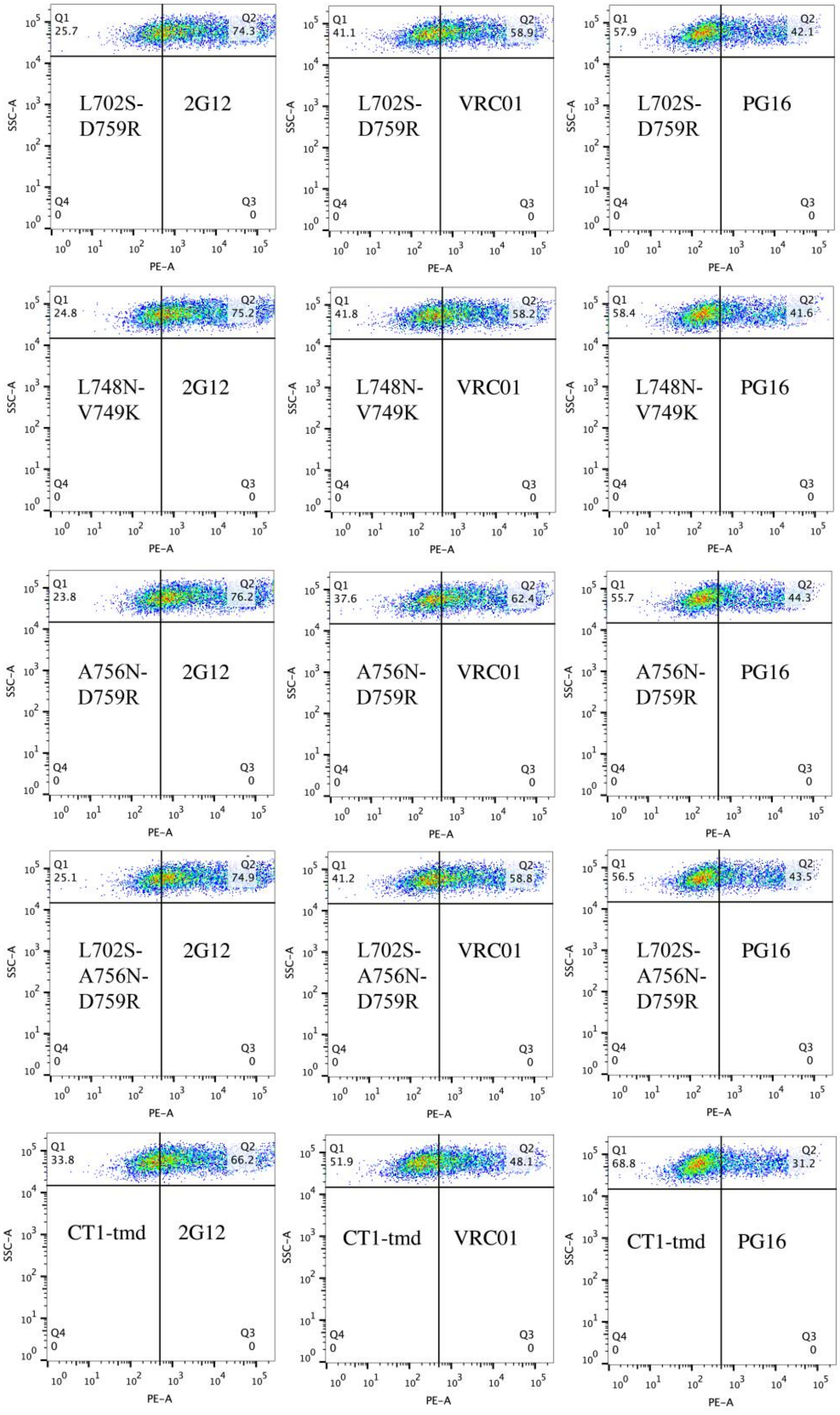

Supplementary Fig. 12 (continue in the next page)

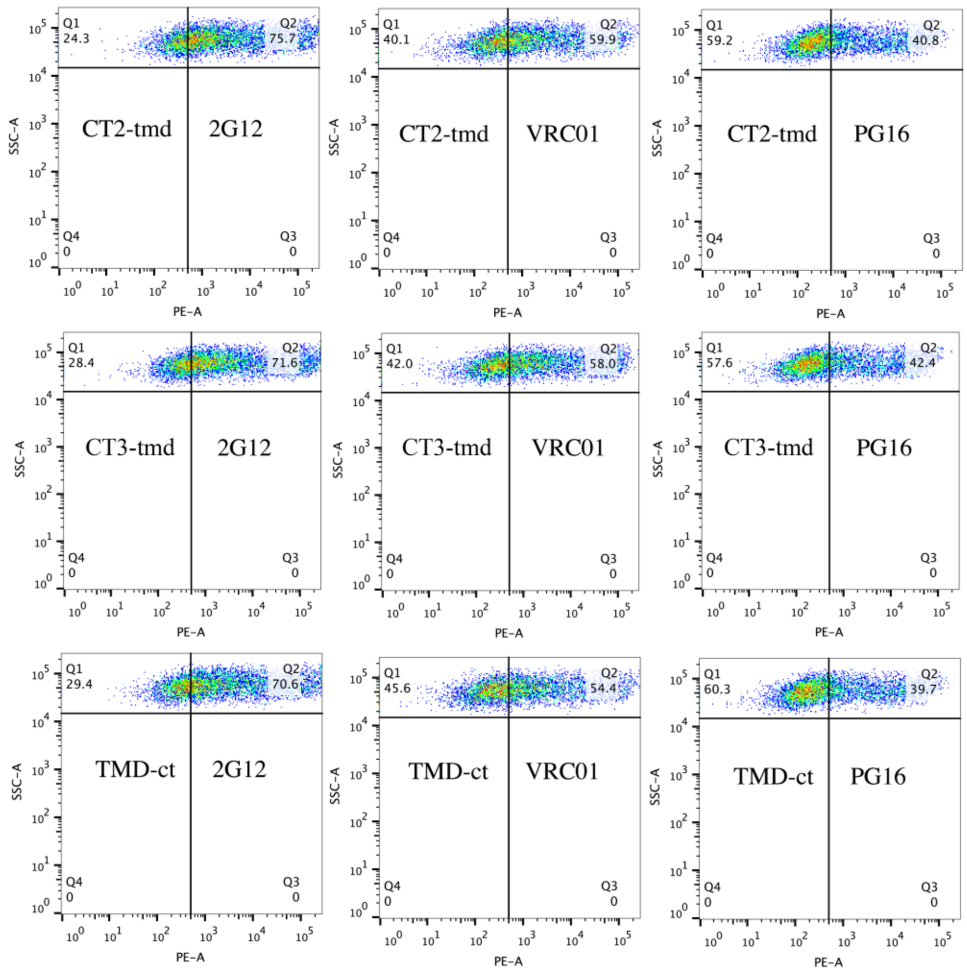

b

Mutation	Mean Fluorescence Intensity (MFI)		
	2G12	VRC01	PG16
WT	7991	5833	5232
V749K	8736	7238	5697
A756N	9405	6825	6217
F774N	8481	6903	5490
L702S-D759R	9287	7567	5440
L748N-V749K	8947	6856	6161
A756N-D759R	10161	8356	6352
L702S-A756N-D759R	9555	7297	6217
CT1-tmd	8170	4706	3157
CT2-tmd	8299	7376	5401
CT3-tmd	8061	7714	5988
TMD-ct	6907	6817	5436
293T	567	73	125

Supplementary Fig. 12 | Cell-surface expression of Env mutants detected by flow cytometry. **a,** Representative dot plots for negative control 293T cells, cells expressing the wildtype Env

(WT), or CT mutants, measured by flow cytometry using monoclonal antibodies 2G12, VRC01 and PG16. **b,** Summary of mean fluorescence intensity (MFI) from data as shown in (a) for the WT and the 11 CT mutants, as well as the 293T cell control.

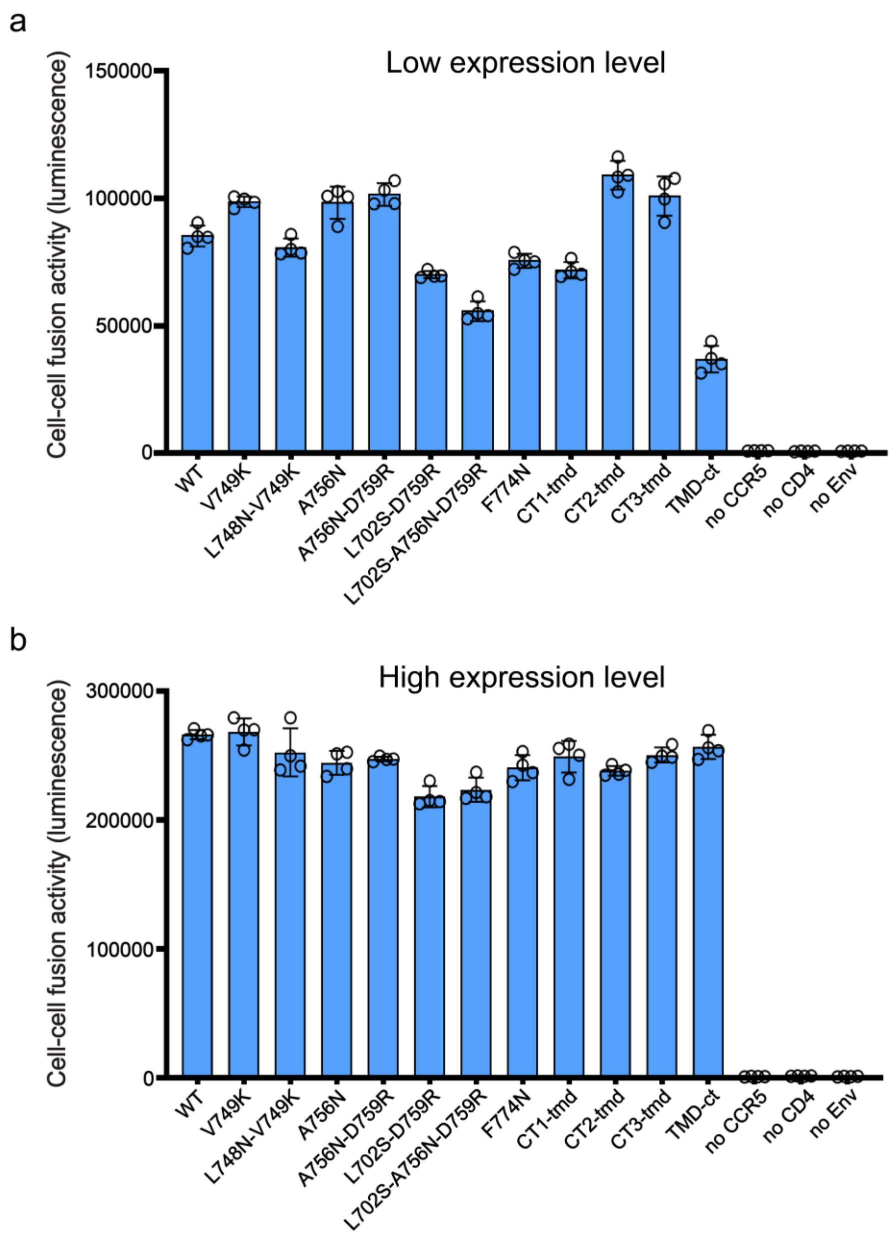

Supplementary Fig. 13 | Cell-cell fusion of HIV-1 Env and its CT mutants at different expression levels. a, Cell-cell fusion of HIV-1 Env and its CT mutants at a low expression level. 293T cells transfected with 75 ng of the 92UG037.8 Env expression plasmid or of each CT mutants were mixed with CD4- and CCR5-expressing cells. Cell-cell fusion led to reconstitution of active β-galactosidase and the fusion activity was quantified by a chemiluminescent assay. **b,** Cell-cell fusion of HIV-1 Env and its CT mutants at a high expression level. 293T cells transfected with 10 μg of the 92UG037.8 Env expression plasmid or of each CT mutants were fused with CD4- and CCR5-expressing cells. In (a) and (b), no Env, no CD4 and no CCR5 were negative controls. The experiments were carried out in triplicate and repeated at least twice with similar results. Error bars indicate the standard deviation calculated by the *Excel* STDEV function. Data points are means ± standard deviations from triplicate measurements. Source data are provided as a Source Data file.

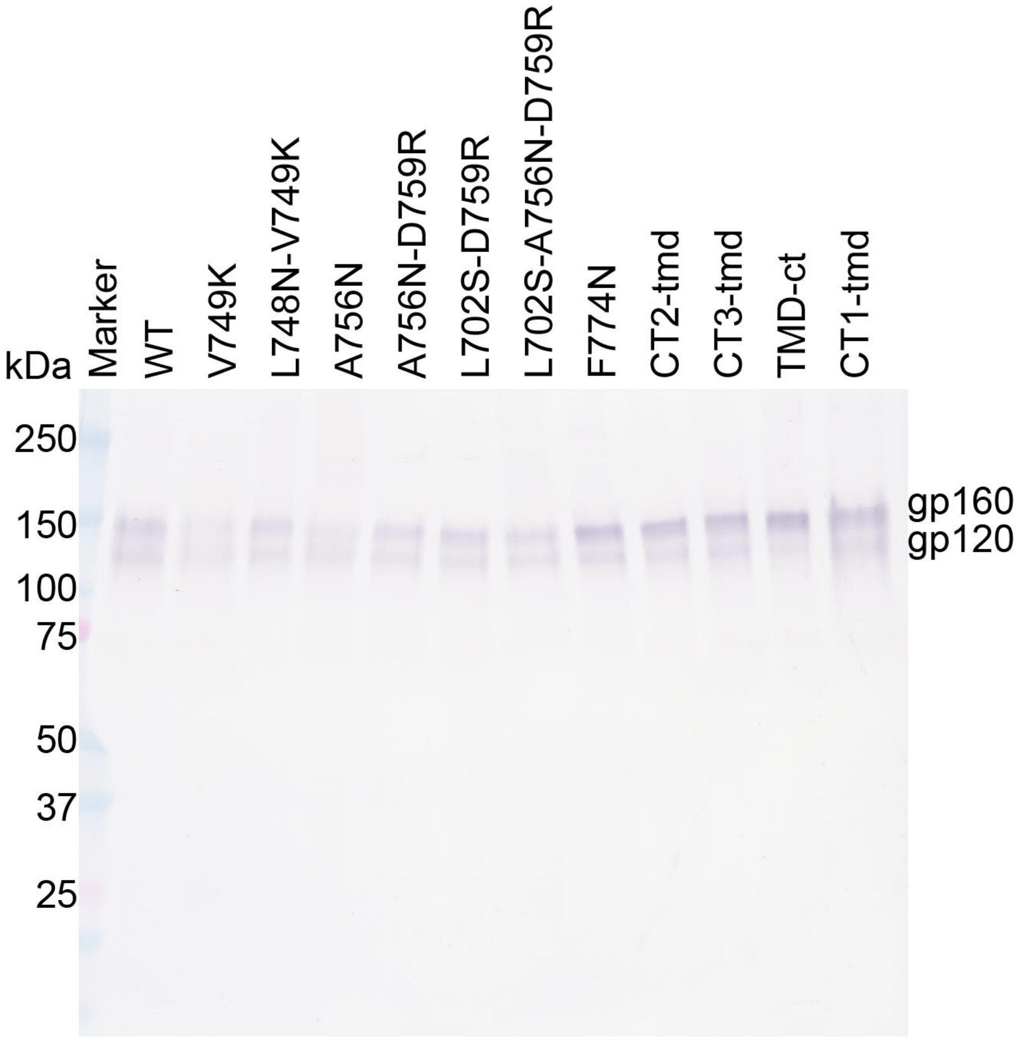

Supplementary Fig. 14 | Incorporation of Env mutants into pseudoviruses analyzed by western blot. Env samples prepared from p24-normalized pseudoviruses containing either HIV-gp92UG037.8 Env or each of its CT mutants were detected by an anti-V3 antibody 3791. The experiment was repeated independently with similar results twice. Source data are provided as a Source Data file.

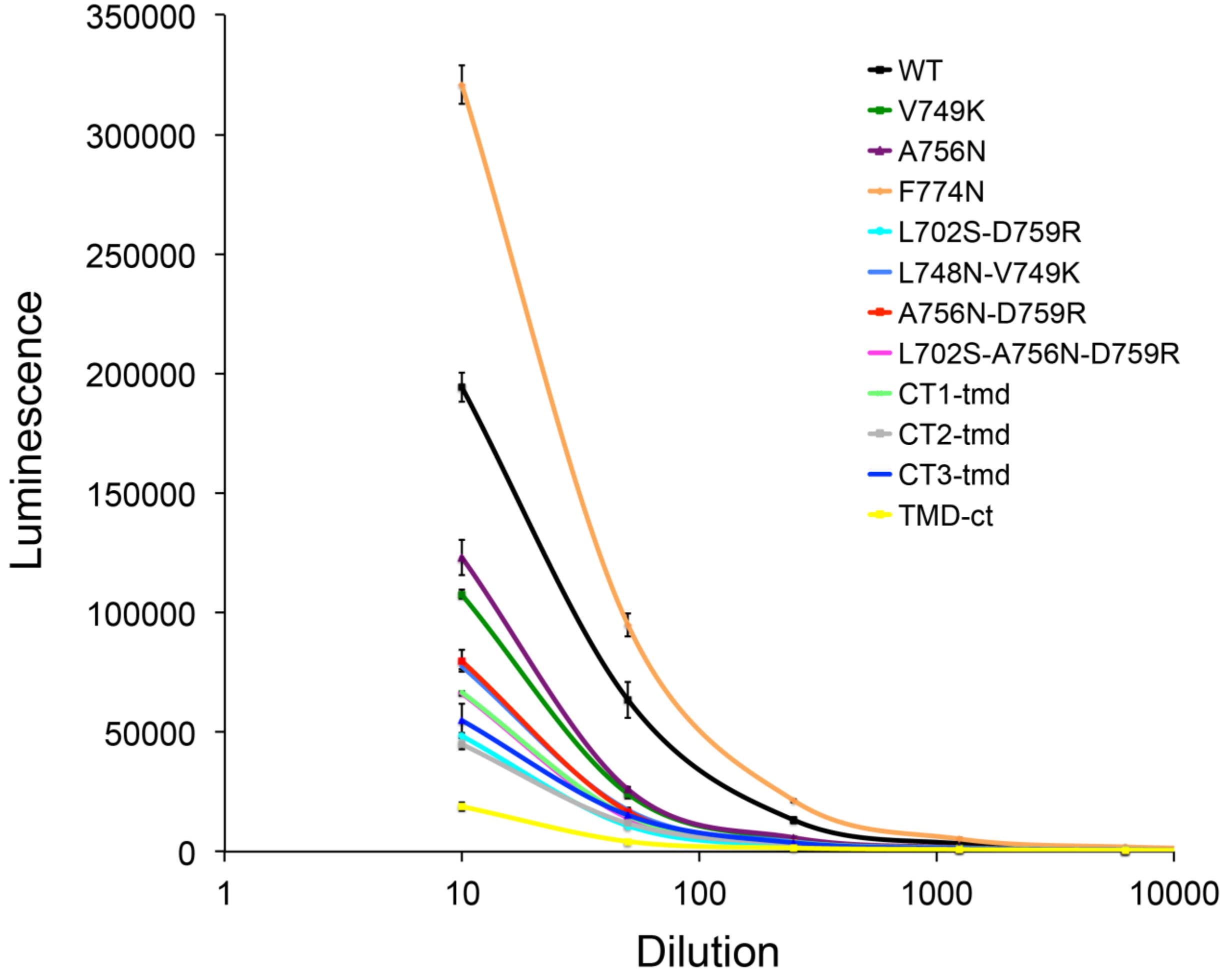

Supplementary Fig. 15 | Viral infectivity of Env mutants. Pseudoviruses containing Env CT mutants were normalized by p24-antigen, titrated 10 times using 5-fold dilution series, and tested for viral infectivity in TZM.bl cells. The experiment was carried out in quadruplicate. Data are presented as mean values and error bars indicate the standard deviation calculated by *Excel*. Source data are provided as a Source Data file.

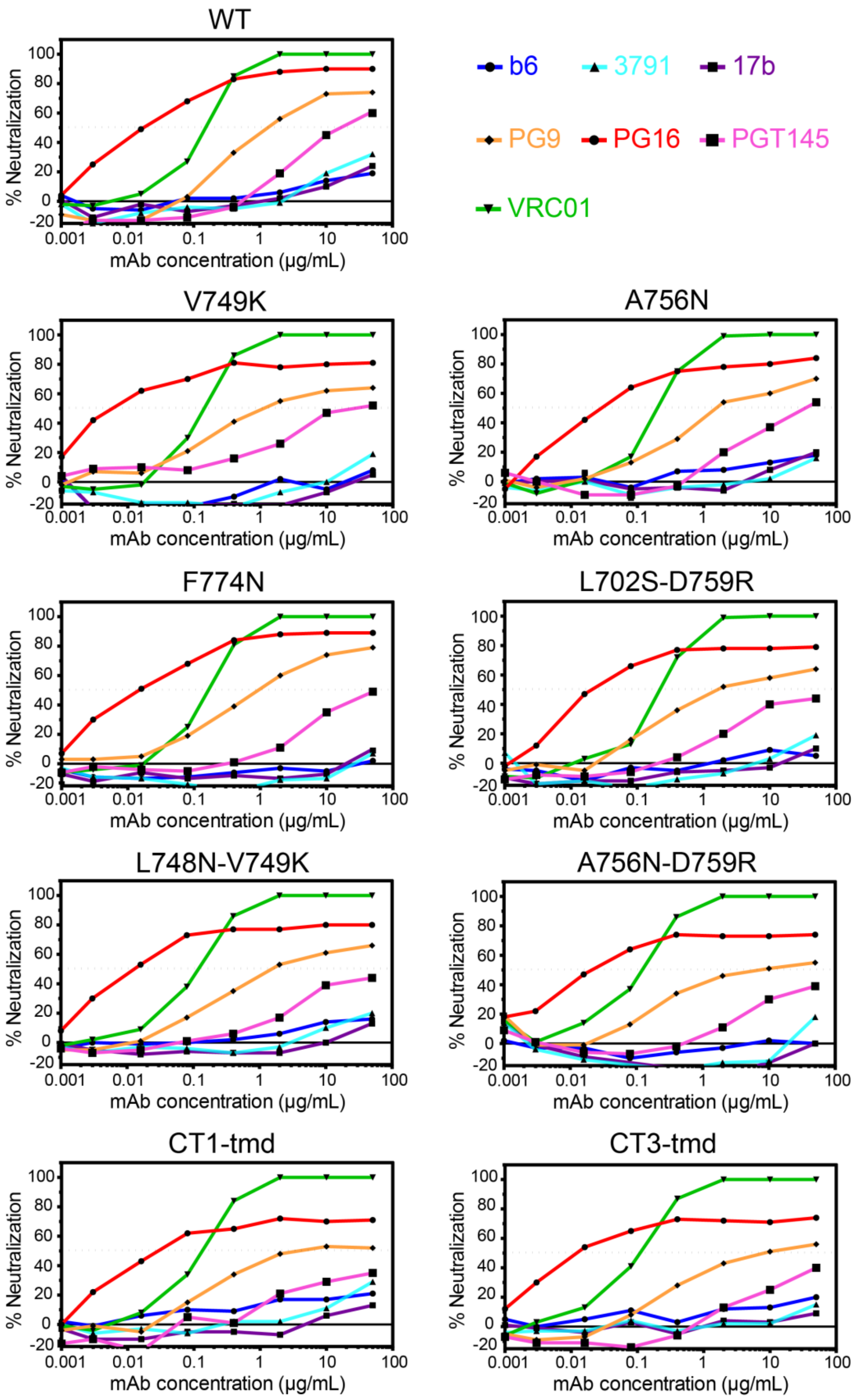

Supplementary Fig. 16 | Effect of mutations in the CT on Env antibody sensitivity. Antibody neutralization of pseudoviruses containing either the 92UG037.8 Env or the CT mutants was

determined for non-neutralizing antibodies, including b6 (CD4 binding site; blue), 3791 (V3; cyan) and 17b (CD4-induced; purple), and trimer-specific bnAbs, including PG9 (orange), PG16 (red) and PGT145 (magenta). The CD4 binding site bnAb VRC01 was a control antibody shown in green. The experiment was performed in duplicate. Source data are provided as a Source Data file.

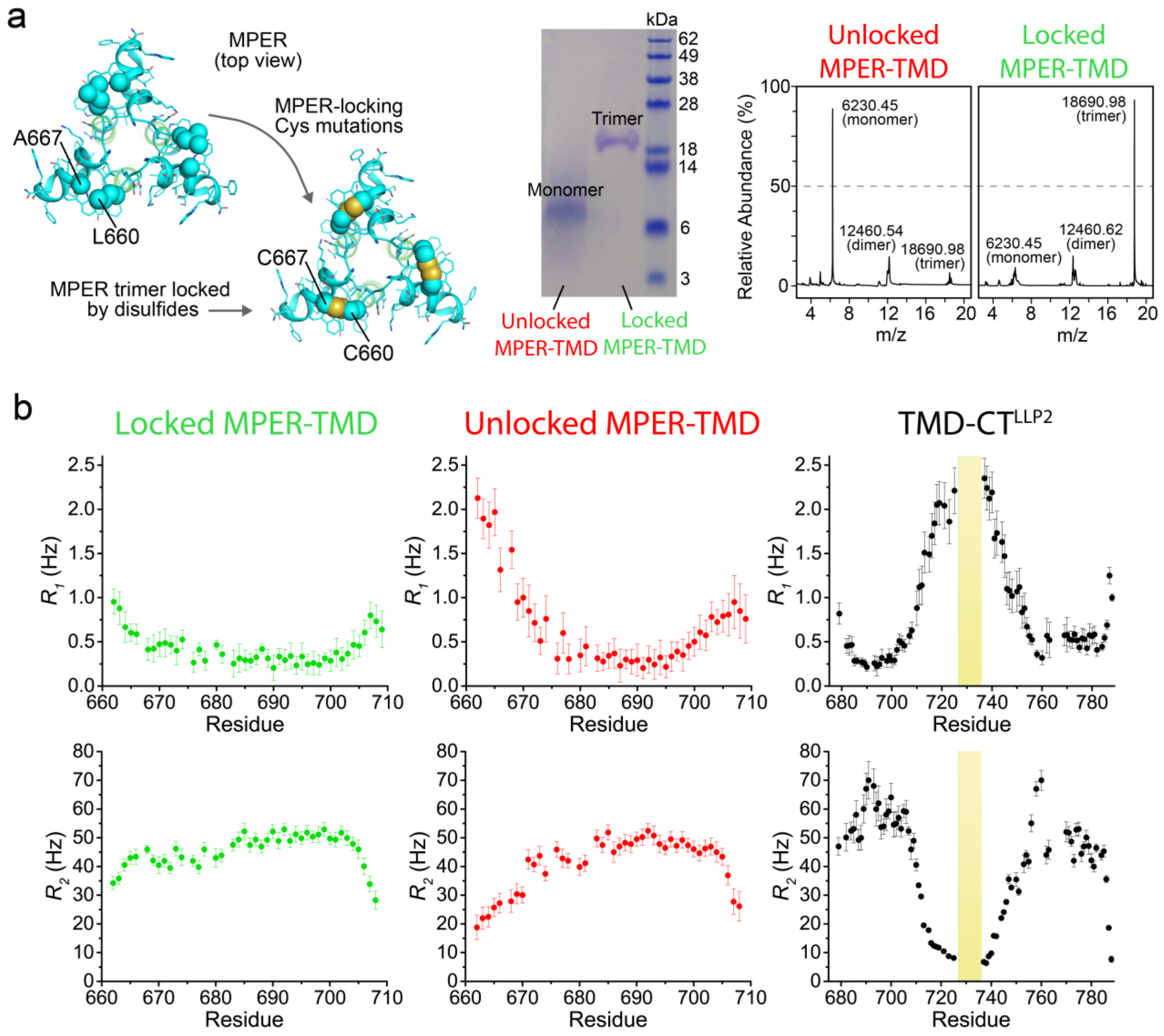

Supplementary Fig. 17 | Locking of the MPER-TMD in the prefusion state and ^{15}N backbone dynamics of the locked MPER-TMD, unlocked MPER-TMD and TMD-CT^{LLP2}. a, Top view of the MPER-TMD trimer with L660 of one chain in close contact with A667 of a neighboring chain (Left). Mutating L660 and A667 to Cys results in disulfide bonds that lock the trimer structure. Urea-PAGE (center) and mass spectrometry (right) analyses of the unlocked (reduced) and locked (oxidized) MPER-TMD mutant showing that it is a covalent trimer when oxidized. The experiment was performed once. **b,** Residue-specific ^{15}N R_1 and R_2 relaxation rates of the locked MPER-TMD (green), unlocked MPER-TMD (red) and TMD-CT^{LLP2} (black). Error bars represent the uncertainty derived from fitting error. Missing data points are due to proline or residues with overlapping peaks. The yellow box marks the deleted residues 726-736 in the TMD-CT^{LLP2} construct. Source data are provided as a Source Data file.

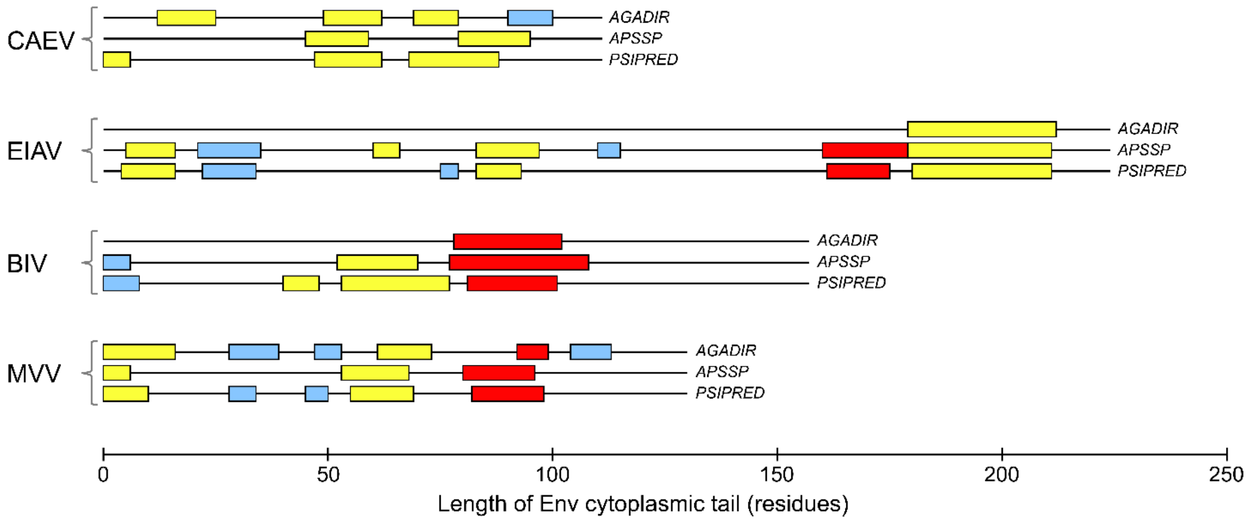

Supplementary Fig. 18 | Relative length of the cytoplasmic tails of fusion proteins from other lentiviruses. For each of the viruses, including the CAEV (caprine arthritis encephalitis virus), EIAV (equine infectious anemia virus), BIV (bovine immunodeficiency virus), and MVV (maedi/visna virus), the cytoplasmic tail (CT) sequence of the viral fusion protein was subject to protein secondary structure prediction using three different programs: *AGADIR*[8], *APSSP*[9], and *PSIPRED*[10]. The sources of the sequences are: UniProtKB D1M7T1 (CAEV), UniProtKB P32541 (EIAV), UniProtKB P19557 (BIV), and UniProtKB P03379) (MVV). The CT sequences are aligned so that the TM helix ends at position 0. The predicted helical segments are represented as boxes colored in red, yellow, and blue coding for hydrophobic, amphipathic or hydrophilic helices, respectively.

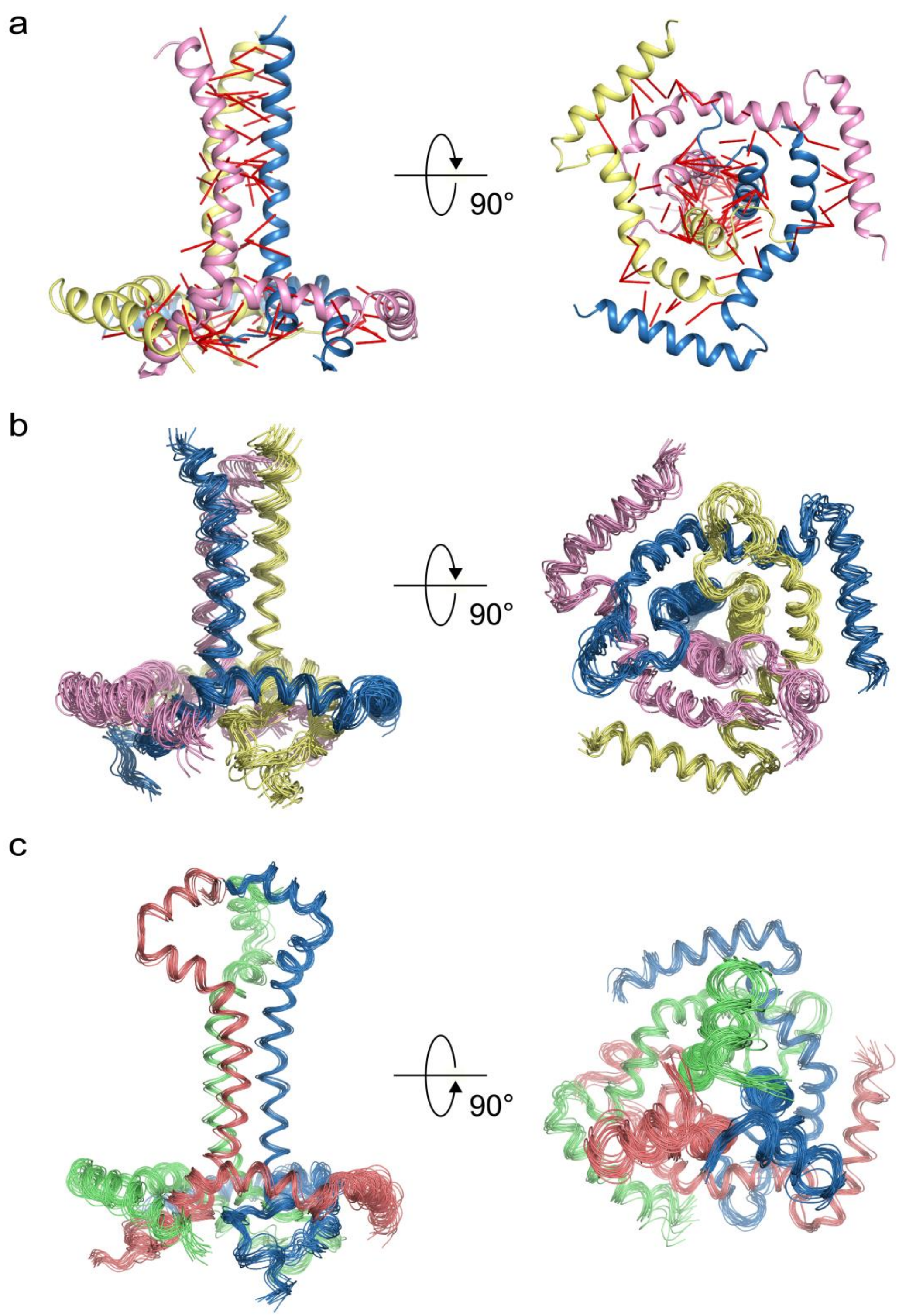

Supplementary Fig. 19 | NMR-derived TMD-CT^{LLP2} and MPER-TMD-CT^{LLP2} structural ensembles. a, Side and bottom view of the TMD-CT^{LLP2} average structure, represented as ribbon, showing NOE-derived inter-chain restraints (red lines). The unstructured KS (residues 711-736) has been omitted for clarity. **b,** Side and bottom view of the ensemble of the 15 lowest-energy TMD-CT^{LLP2} structures calculated using NMR-derived structural restraints (Supplementary Table

11). Structures are represented as thin ribbons and Residues 726-736 have been omitted for clarity. **c,** Side and top view of the NMR ensemble of the 15 lowest-energy MPER-TMD-CT^{LLP2} structures calculated using NMR-derived structural restraints (Supplementary Table 12). Structures are represented as thin ribbons and residues 726-736 have been omitted.

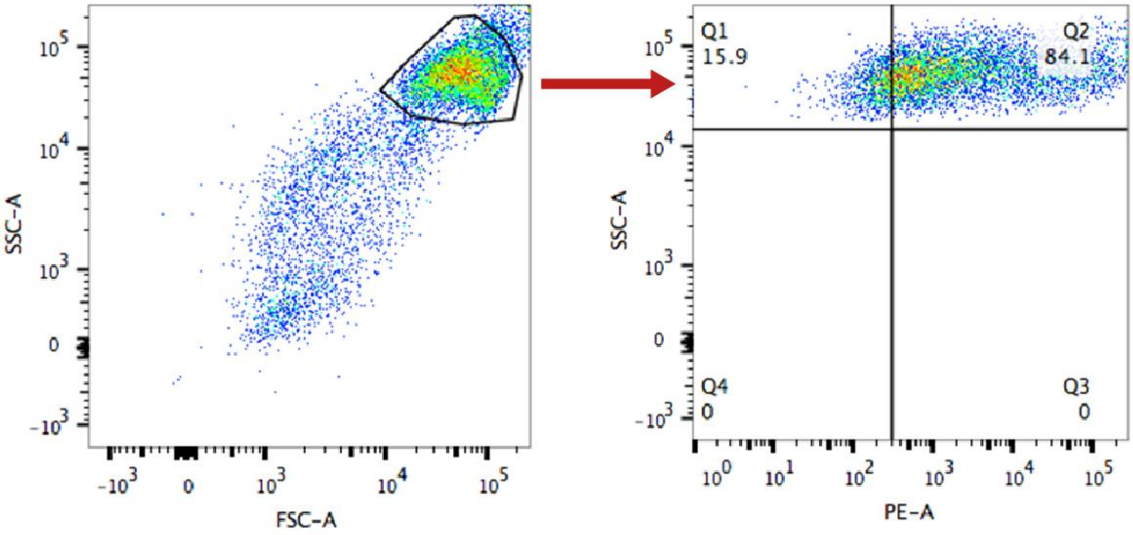

Supplementary Fig. 20 | Flow cytometry gating strategy.

Supplementary Table 1 | List of CT mutants. Mutants (#1-21) are named borrowing the residue type and number from the 92UG037.8 and 92UG024.2 sequence, respectively. Mutants (#22-28) are named indicating the site of the mutation and the interface affected in uppercase and lowercase, respectively. The mutants that have been thoroughly characterized are highlighted in yellow.

Mutant	#	Description		Control exps.
		92UG037.8 sequence (functional data)	92UG024.2 sequence (NMR constructs)	
**L702Q**	1	L697Q	L702Q	
**L702S**	3	L697S	L702S	
**I746K**	7	I741K	I746K	
**R747D**	5	R742D	R742D	
**L748N**	2	L743N	L748N	
**L748Q**	4	L743Q	L748Q	
**V749K**	6	V744K	V749K	✓
**G751Y**	8	G746Y	G751Y	
**L755N**	9	L750N	L755N	
**A756N**	10	A751N	I756N	✓
**D758R**	11	D753R	D758R	
**D759R**	12	D754R	D759R	
**L771K**	13	L766K	L771K	
**F774N**	14	F669N	L774N	✓
**R780D**	15	R775D	R780D	
**L702S-D759R**	16	L697S-D754R	L702S- D759R	✓
**L748N-V749K**	17	L743N-V744K	L748N- V749K	✓
**A756N-D759R**	18	A751N-D754R	I756N- D759R	✓
**L771K-F774N**	19	L766K-F769N	L771K-L774N	
**L702S-A756N-D759R**	20	L697S-A751N-D754R	L702S-I756N-D759R	✓
**A756N-L760S-L763N-C764E**	21	A751N-L755S-L758N-C759E	I756N-L760S-L763N-C764E	
**polyA(748-762)**	22	polyA(743-757)	polyA(748-762)	
**Δ(748-762)**	23	Δ(743-757)	Δ(748-762)	
**CT1-tmd**	24	I741N-L743S-V744R-L750A-D753K-D754R	I746N-L748S-V749R-L755A-D758K-D759R	✓
**CT2-tmd**	25	L743S-L750S-D753A-D754A-N757A	L748S-L755S-D758A-D759A-S762A	✓
**CT3-tmd**	26	I741S-L743S-V744S-L750S-D753A-D754A-N757A	I746S-L748S-V749S-L755S-D758A-D759A-S762A	✓
**CT4-tmd**	27	I741N-L743S-V744R-L750A-A751N-D753K-D754R-L755S-L758N-C759E	I746N-L748S-V749R-L755A-I756N-D758K-D759R-L760S-L763N-C764E	
**TMD-ct**	28	V699S-I700S-N701A-V703S-R704A-Q705A	L704S-V705S-N706A-V708S-R709A-Q710A	✓

Supplementary Table 2 | Antibody neutralization of CT mutants.

Titer in TZM.bl Cells (µg/mL)											
Mutant		TCID50	b6 IgG			17b			3791		
**WT**		69,877	IC50	IC80	MPI	IC50	IC80	MPI	IC50	IC80	MPI
			>50	>50	19	>50	>50	24	>50	>50	32
VRC01			PG9			PG16			PG145		
IC50	IC80	MPI	IC50	IC80	MPI	IC50	IC80	MPI	IC50	IC80	MPI
0.143	0.318	100	1.075	>50	74	0.017	0.210	90	18.278	>50	61
Mutant		TCID50	b6 IgG			17b			3791		
**L702Q**		18,275	IC50	IC80	MPI	IC50	IC80	MPI	IC50	IC80	MPI
			>50	>50	4	>50	>50	8	>50	>50	0
VRC01			PG9			PG16			PG145		
IC50	IC80	MPI	IC50	IC80	MPI	IC50	IC80	MPI	IC50	IC80	MPI
0.226	0.599	100	2.508	>50	65	0.034	1.070	82	>50	>50	43
Mutant		TCID50	b6 IgG			17b			3791		
**L702S**		69,877	IC50	IC80	MPI	IC50	IC80	MPI	IC50	IC80	MPI
			>50	>50	4	>50	>50	12	>50	>50	14
VRC01			PG9			PG16			PG145		
IC50	IC80	MPI	IC50	IC80	MPI	IC50	IC80	MPI	IC50	IC80	MPI
0.215	0.722	100	1.521	>50	74	0.032	0.277	88	31.473	>50	53
Mutant		TCID50	b6 IgG			17b			3791		
**I746K**		349,386	IC50	IC80	MPI	IC50	IC80	MPI	IC50	IC80	MPI
			>50	>50	23	>50	>50	25	>50	>50	34
VRC01			PG9			PG16			PG145		
IC50	IC80	MPI	IC50	IC80	MPI	IC50	IC80	MPI	IC50	IC80	MPI
0.115	0.320	100	1.012	>50	69	0.013	0.462	84	24.266	>50	57
Mutant		TCID50	b6 IgG			17b			3791		
**R747D**		18,275	IC50	IC80	MPI	IC50	IC80	MPI	IC50	IC80	MPI
			>50	>50	0	>50	>50	0	>50	>50	2
VRC01			PG9			PG16			PG145		
IC50	IC80	MPI	IC50	IC80	MPI	IC50	IC80	MPI	IC50	IC80	MPI
0.140	0.374	100	0.854	>50	72	0.013	0.219	87	46.006	>50	52
Mutant		TCID50	b6 IgG			17b			3791		
**L748N**		53,437	IC50	IC80	MPI	IC50	IC80	MPI	IC50	IC80	MPI
			>50	>50	3	>50	>50	0	>50	>50	23
VRC01			PG9			PG16			PG145		
IC50	IC80	MPI	IC50	IC80	MPI	IC50	IC80	MPI	IC50	IC80	MPI
0.113	0.266	100	1.058	>50	72	0.011	0.573	84	31.535	>50	57

Supplementary Table 2 (continued)

Mutant		TCID50	b6 IgG			17b			3791		
**L748Q**		781,250	IC50	IC80	MPI	IC50	IC80	MPI	IC50	IC80	MPI
			>50	>50	17	>50	>50	12	>50	>50	17
VRC01			PG9			PG16			PG145		
IC50	IC80	MPI	IC50	IC80	MPI	IC50	IC80	MPI	IC50	IC80	MPI
0.129	0.356	100	0.767	>50	76	0.010	0.233	87	33.764	>50	53
Mutant		TCID50	b6 IgG			17b			3791		
**V749K**		13,975	IC50	IC80	MPI	IC50	IC80	MPI	IC50	IC80	MPI
			>50	>50	8	>50	>50	5	>50	>50	19
VRC01			PG9			PG16			PG145		
IC50	IC80	MPI	IC50	IC80	MPI	IC50	IC80	MPI	IC50	IC80	MPI
0.135	0.297	100	0.964	>50	64	0.006	1.700	81	20.588	>50	52
Mutant		TCID50	b6 IgG			17b			3791		
**G751Y**		69,877	IC50	IC80	MPI	IC50	IC80	MPI	IC50	IC80	MPI
			>50	>50	3	>50	>50	3	>50	>50	18
VRC01			PG9			PG16			PG145		
IC50	IC80	MPI	IC50	IC80	MPI	IC50	IC80	MPI	IC50	IC80	MPI
0.096	0.258	100	1.318	>50	66	0.008	>50	76	>50	>50	48
Mutant		TCID50	b6 IgG			17b			3791		
**L755N**		6,250	IC50	IC80	MPI	IC50	IC80	MPI	IC50	IC80	MPI
			>50	>50	21	>50	>50	11	>50	>50	34
VRC01			PG9			PG16			PG145		
IC50	IC80	MPI	IC50	IC80	MPI	IC50	IC80	MPI	IC50	IC80	MPI
0.106	0.225	100	1.444	>50	66	0.014	0.884	83	12.172	>50	51
Mutant		TCID50	b6 IgG			17b			3791		
**A756N**		13,975	IC50	IC80	MPI	IC50	IC80	MPI	IC50	IC80	MPI
			>50	>50	18	>50	>50	20	>50	>50	16
VRC01			PG9			PG16			PG145		
IC50	IC80	MPI	IC50	IC80	MPI	IC50	IC80	MPI	IC50	IC80	MPI
0.189	0.503	100	1.835	>50	70	0.028	11.168	84	32.972	>50	54
Mutant		TCID50	b6 IgG			17b			3791		
**D758R**		13,975	IC50	IC80	MPI	IC50	IC80	MPI	IC50	IC80	MPI
			>50	>50	7	>50	>50	5	>50	>50	23
VRC01			PG9			PG16			PG145		
IC50	IC80	MPI	IC50	IC80	MPI	IC50	IC80	MPI	IC50	IC80	MPI
0.088	0.294	100	0.801	>50	70	0.009	0.491	83	26.853	>50	52

Supplementary Table 2 (continued)

Mutant		TCID50	b6 IgG			17b			3791		
**D759R**		2,795	IC50	IC80	MPI	IC50	IC80	MPI	IC50	IC80	MPI
			>50	>50	5	>50	>50	0	>50	>50	27
VRC01			PG9			PG16			PG145		
IC50	IC80	MPI	IC50	IC80	MPI	IC50	IC80	MPI	IC50	IC80	MPI
0.115	0.248	100	0.920	>50	66	0.014	0.424	79	49.309	>50	50
Mutant		TCID50	b6 IgG			17b			3791		
**L771K**		53,437	IC50	IC80	MPI	IC50	IC80	MPI	IC50	IC80	MPI
			>50	>50	0	>50	>50	0	>50	>50	14
VRC01			PG9			PG16			PG145		
IC50	IC80	MPI	IC50	IC80	MPI	IC50	IC80	MPI	IC50	IC80	MPI
0.092	0.249	100	0.672	>50	77	0.009	0.080	88	28.761	>50	55
Mutant		TCID50	b6 IgG			17b			3791		
**F774N**		1,746,928	IC50	IC80	MPI	IC50	IC80	MPI	IC50	IC80	MPI
			>50	>50	2	>50	>50	10	>50	>50	7
VRC01			PG9			PG16			PG145		
IC50	IC80	MPI	IC50	IC80	MPI	IC50	IC80	MPI	IC50	IC80	MPI
0.152	0.409	100	0.742	>50	79	0.015	0.200	89	>50	>50	49
Mutant		TCID50	b6 IgG			17b			3791		
**R780D**		18,275	IC50	IC80	MPI	IC50	IC80	MPI	IC50	IC80	MPI
			>50	>50	1	>50	>50	12	>50	>50	24
VRC01			PG9			PG16			PG145		
IC50	IC80	MPI	IC50	IC80	MPI	IC50	IC80	MPI	IC50	IC80	MPI
0.082	0.304	100	0.573	>50	78	0.007	0.095	90	15.588	>50	61
Mutant		TCID50	b6 IgG			17b			3791		
**L702S-D759R**		3,655	IC50	IC80	MPI	IC50	IC80	MPI	IC50	IC80	MPI
			>50	>50	5	>50	>50	10	>50	>50	19
VRC01			PG9			PG16			PG145		
IC50	IC80	MPI	IC50	IC80	MPI	IC50	IC80	MPI	IC50	IC80	MPI
0.205	0.541	100	1.153	>50	64	0.021	>50	79	>50	>50	44
Mutant		TCID50	b6 IgG			17b			3791		
**L748N-V749K**		267,184	IC50	IC80	MPI	IC50	IC80	MPI	IC50	IC80	MPI
			>50	>50	16	>50	>50	13	>50	>50	20
VRC01			PG9			PG16			PG145		
IC50	IC80	MPI	IC50	IC80	MPI	IC50	IC80	MPI	IC50	IC80	MPI
0.111	0.303	100	1.647	>50	66	0.013	36.106	80	>50	>50	44

Supplementary Table 2 (continued)

Mutant		TCID50	b6 IgG			17b			3791		
**A756N-D759R**		13,975	IC50	IC80	MPI	IC50	IC80	MPI	IC50	IC80	MPI
			>50	>50	0	>50	>50	0	>50	>50	18
VRC01			PG9			PG16			PG145		
IC50	IC80	MPI	IC50	IC80	MPI	IC50	IC80	MPI	IC50	IC80	MPI
0.102	0.294	100	3.277	>50	55	0.023	>50	74	>50	>50	39
Mutant		TCID50	b6 IgG			17b			3791		
**L771K-F774N**		18,275	IC50	IC80	MPI	IC50	IC80	MPI	IC50	IC80	MPI
			>50	>50	8	>50	>50	7	>50	>50	20
VRC01			PG9			PG16			PG145		
IC50	IC80	MPI	IC50	IC80	MPI	IC50	IC80	MPI	IC50	IC80	MPI
0.104	0.284	100	0.504	39.024	82	0.005	0.108	91	19.283	>50	62
Mutant		TCID50	b6 IgG			17b			3791		
**L702S-A756N-D759R**		10,687	IC50	IC80	MPI	IC50	IC80	MPI	IC50	IC80	MPI
			>50	>50	12	>50	>50	6	>50	>50	16
VRC01			PG9			PG16			PG145		
IC50	IC80	MPI	IC50	IC80	MPI	IC50	IC80	MPI	IC50	IC80	MPI
0.140	0.310	100	>50	>50	48	0.018	>50	67	>50	>50	30
Mutant		TCID50	b6 IgG			17b			3791		
**A756N-L760S-L763N-C764E**		1,250	IC50	IC80	MPI	IC50	IC80	MPI	IC50	IC80	MPI
			>50	>50	1	>50	>50	0	>50	>50	17
VRC01			PG9			PG16			PG145		
IC50	IC80	MPI	IC50	IC80	MPI	IC50	IC80	MPI	IC50	IC80	MPI
0.085	0.219	100	0.280	7.796	83	0.006	0.044	91	7.238	>50	64
Mutant		TCID50	b6 IgG			17b			3791		
**polyA(748-762)**		13,975	IC50	IC80	MPI	IC50	IC80	MPI	IC50	IC80	MPI
			>50	>50	12	>50	>50	15	>50	>50	32
VRC01			PG9			PG16			PG145		
IC50	IC80	MPI	IC50	IC80	MPI	IC50	IC80	MPI	IC50	IC80	MPI
0.104	0.295	100	1.266	>50	72	0.011	0.545	83	33.704	>50	56
Mutant		TCID50	b6 IgG			17b			3791		
**Δ(748-762)**		91,376	IC50	IC80	MPI	IC50	IC80	MPI	IC50	IC80	MPI
			>50	>50	20	>50	>50	17	>50	>50	24
VRC01			PG9			PG16			PG145		
IC50	IC80	MPI	IC50	IC80	MPI	IC50	IC80	MPI	IC50	IC80	MPI
0.108	0.298	100	1.406	>50	70	0.011	1.220	83	>50	>50	50

Supplementary Table 2 (continued)

Mutant		TCID50	b6 IgG			17b			3791		
**CT1-tmd**		13,975	IC50	IC80	MPI	IC50	IC80	MPI	IC50	IC80	MPI
			>50	>50	21	>50	>50	13	>50	>50	29
VRC01			PG9			PG16			PG145		
IC50	IC80	MPI	IC50	IC80	MPI	IC50	IC80	MPI	IC50	IC80	MPI
0.126	0.342	100	2.533	>50	52	0.027	>50	71	>50	>50	35
Mutant		TCID50	b6 IgG			17b			3791		
**CT2-tmd**		53,437	IC50	IC80	MPI	IC50	IC80	MPI	IC50	IC80	MPI
			>50	>50	20	>50	>50	9	>50	>50	23
VRC01			PG9			PG16			PG145		
IC50	IC80	MPI	IC50	IC80	MPI	IC50	IC80	MPI	IC50	IC80	MPI
0.095	0.266	100	46.920	>50	51	0.063	>50	66	>50	>50	37
Mutant		TCID50	b6 IgG			17b			3791		
**CT3-tmd**		91,376	IC50	IC80	MPI	IC50	IC80	MPI	IC50	IC80	MPI
			>50	>50	20	>50	>50	9	>50	>50	15
VRC01			PG9			PG16			PG145		
IC50	IC80	MPI	IC50	IC80	MPI	IC50	IC80	MPI	IC50	IC80	MPI
0.088	0.308	100	8.109	>50	56	0.012	>50	74	>50	>50	40
Mutant		TCID50	b6 IgG			17b			3791		
**CT4-tmd**		13,975	IC50	IC80	MPI	IC50	IC80	MPI	IC50	IC80	MPI
			>50	>50	11	>50	>50	10	>50	>50	26
VRC01			PG9			PG16			PG145		
IC50	IC80	MPI	IC50	IC80	MPI	IC50	IC80	MPI	IC50	IC80	MPI
0.085	0.247	100	0.322	6.742	84	0.007	0.095	91	8.695	>50	60
Mutant		TCID50	b6 IgG			17b			3791		
**TMD-ct**		13,975	IC50	IC80	MPI	IC50	IC80	MPI	IC50	IC80	MPI
			1.148	>50	60	>50	>50	45	1.208	>50	70
VRC01			PG9			PG16			PG145		
IC50	IC80	MPI	IC50	IC80	MPI	IC50	IC80	MPI	IC50	IC80	MPI
0.056	0.156	100	>50	>50	0	>50	>50	2	>50	>50	0

Supplementary Table 3 | Chronic HIV+ serum samples for tier phenotyping.

Titer in TZM.bl Cells (μg/mL)												
**Sample ID**	**WT**			**CT2-tmd**			**CT3-tmd**			**TMD-ct**		
	IC50	IC80	MPI	IC50	IC80	MPI	IC50	IC80	MPI	IC50	IC80	MPI
HIV-018	<20	<20	22	<20	<20	48	<20	<20	43	335	28	85
HIV-019	31	<20	60	21	<20	51	28	<20	59	155	29	85
HIV-021	<20	<20	6	<20	<20	26	<20	<20	29	89	<20	80
HIV-023	<20	<20	0	<20	<20	0	<20	<20	0	<20	<20	44
HIV-024	486	179	97	821	264	99	986	313	98	1,776	500	100
HIV-025	<20	<20	0	<20	<20	13	<20	<20	20	227	39	85
HIV-026	<20	<20	0	<20	<20	0	<20	<20	0	90	<20	71

Supplementary Table 4 | Additional bnAbs epitope phenotyping.

Titer in TZM.bl Cells (µg/mL)												
**bnAbs (epitope)**	**WT**			**CT2-tmd**			**CT3-tmd**			**TMD-ct**		
	IC50	IC80	MPI	IC50	IC80	MPI	IC50	IC80	MPI	IC50	IC80	MPI
3BNC117 (CD4bs)	0.015	0.044	100	0.010	0.029	100	0.008	0.030	100	0.005	0.025	100
PGT121 (V3-glycan)	0.097	0.266	100	0.058	0.202	100	0.052	0.185	100	0.052	0.147	100
10-1074 (V3-glycan)	0.101	0.331	100	0.100	0.342	100	0.072	0.241	100	0.096	0.318	100
PGDM1400 (V3-glycan)	0.103	0.347	99	0.066	0.231	99	0.063	0.221	99	0.061	0.318	96
PGT151 (gp120/gp41 interface)	>50	>50	14	>50	>50	14	>50	>50	24	>50	>50	0
8ANC194 (gp120/gp41 interface)	1.044	4.636	98	0.697	3.357	98	0.537	3.749	98	0.545	1.893	99
4E10 (MPER)	0.467	3.012	99	0.046	0.289	100	0.049	0.424	100	0.031	0.186	100

Supplementary Table 5 | Amino acid sequence of the NMR and OG-label constructs used in this study. The gp41 sequences are based on that of clade D HIV-1 isolate 92UG024.2. Residues of the MPER, TMD-KS and CT^{LLP2} are shown in blue, red and green, respectively. The 10 residues of the KS that were deleted are show in purple. Residues highlighted in bold and black indicate the relevant modifications introduced to each construct.

**WT gp41** (residues 677-788)	NWLWYIRIFIIIVGSLIGLRIVFAVLSLVNRVRQGYSPLSFQTHLPTPRGPDRP EGIEEEGGERDRDRSIRLVNGSLALIWDDLRSLCLFSYHRLRDLLLIVTRIVE LLGRR
NMR constructs	
**TMD-CT^{LLP2}** (residues 677-725; 737-788)	NWLWYIRIFIIIVGSLIGLRIVFAVLSLVNRVRQGYSPLSFQTHLPTPRGGERD RDRSIRLVNGSLALIWDDLRSL**S**LFSYHRLRDLLLIVTRIVELLGRR<br>Note: residues 726-736 were deleted; C764S
**TMD-CT^{LLP2} G738C**	NWLWYIRIFIIIVGSLIGLRIVFAVLSLVNRVRQGYSPLSFQTHLPTPRG**C**ERD RDRSIRLVNGSLALIWDDLRSLSLFSYHRLRDLLLIVTRIVELLGRR
**TMD-CT^{LLP2} S764C**	NWLWYIRIFIIIVGSLIGLRIVFAVLSLVNRVRQGYSPLSFQTHLPTPRGGERD RDRSIRLVNGSLALIWDDLRSL**C**LFSYHRLRDLLLIVTRIVELLGRR<br>Note: the native Cys was reintroduced
**TMD-CT^{LLP2} C789**	NWLWYIRIFIIIVGSLIGLRIVFAVLSLVNRVRQGYSPLSFQTHLPTPRGGERD RDRSIRLVNGSLALIWDDLRSL<u>S</u>LFSYHRLRDLLLIVTRIVELLGRR**C**
**TMD-KS** (residues 677-725)	NWLWYIRIFIIIVGSLIGLRIVFAVLSLVNRVRQGYSPLSFQTHLPTPR**M**<br>Note: the additional C-terminal Met was introduced to allow CNBr cleavage of TMD-KS from CT^{LLP2} during protein purification
**CT^{LLP2}** (residues 737-788)	GGERDRDRSIRLVNGSLALIWDDLRSLSLFSYHRLRDLLLIVTRIVELLGRR
**CT^{LLP2} C789**	GGERDRDRSIRLVNGSLALIWDDLRSLSLFSYHRLRDLLLIVTRIVELLGRR**C**
**CT2-tmd**	NWLWYIRIFIIIVGSLIGLRIVFAVLSLVNRVRQGYSPLSFQTHLPTPRGGERD RDRSIR**S**VNGSLA**S**IW**AA**LR**A**LSLFSYHRLRDLLLIVTRIVELLGRR<br>Note: L748S-L755S-D758A-D759A-S762A mutations were introduced
**CT2-tmd S764C**	NWLWYIRIFIIIVGSLIGLRIVFAVLSLVNRVRQGYSPLSFQTHLPTPRGGERD RDRSIR**S**VNGSLA**S**IW**AA**LR**A**L**C**LFSYHRLRDLLLIVTRIVELLGRR<br>Note: the native Cys was reintroduced to the TMD-CT^{LLP2} CT2-tmd
**MPER-TMD L660C A667C** (residues 660-710)	**C**LELDKW**C**SLWNWFDITNWLWYIRIFIIIVGSLIGLRIVFAVLSLVNRVRQ<br>Note: the MPER was locked or unlocked controlling on the oxidation state of the Cys
OG-label constructs	
**His$_6$-tagged TMD-CT^{LLP2}**	NWLWYIRIFIIIVGSLIGLRIVFAVLSLVNRVRQGYSPLSFQTHLPTPRGGERD RDRSIRLVNGSLALIWDDLRSLSLFSYHRLRDLLLIVTRIVELLGRR**HHHHH H**
**His$_6$-tagged CT^{LLP2}**	GGERDRDRSIRLVNGSLALIWDDLRSLSLFSYHRLRDLLLIVTRIVELLGRR **HHHHHH**

Supplementary Table 6 | DNA sequence of the synthetic genes and primers used in this study.

	Synthetic genes
**#1**	AATTGGCTGTGGTATATTCGTATTTTCATTATCATAGTGGGAAGCCTGATTGGCCTGCGTATTG<br>TGTTTGCGGTTCTGAGCCTGGTGAATCGGGTGCGTCAGGGTTATAGCCCGCTGAGCTTTCAGAC<br>CCATCTGCCTACCCCGCGTGGCCCGGATCGTCCGGAAGGGATAGAAGAAGAGGGCGGAGAAC<br>GCGACCGTGATCGTAGCATTCGTCTGGTTAATGGCAGCCTGGCACTGATTTGGGATGATCTGCG<br>TAGCCTGTGCCTTTTTAGCTATCATCGTCTGCGTGATTTACTGCTGATTGTGACCCGTATCGTGG<br>AACTGCTGGGCCGTCGT
**#2**	AATTGGCTGTGGTATATTCGTATTTTCATTATCATAGTGGGAAGCCTGATTGGCCTGCGTATTG<br>TGTTTGCGGTTCTGAGCCTGGTGAATCGGGTGCGTCAGGGTTATAGCCCGCTGAGCTTTCAGAC<br>CCATCTGCCTACCCCGCGTGGCTGCGAACGCGACCGTGATCGTAGCATTCGTCTGGTTAATGGC<br>AGCCTGGCACTGATTTGGGATGATCTGCGTAGCCTGAGCCTTTTTAGCTATCATCGTCTGCGTG<br>ATTTACTGCTGATTGTGACCCGTATCGTGGAACTGCTGGGCCGTCGT
**#3**	AATTGGCTGTGGTATATTCGTATTTTCATTATCATAGTGGGAAGCCTGATTGGCCTGCGTATTG<br>TGTTTGCGGTTCTGAGCCTGGTGAATCGGGTGCGTCAGGGTTATAGCCCGCTGAGCTTTCAGAC<br>CCATCTGCCTACCCCGCGTATGGGCGGAGAACGCGACCGTGATCGTAGCATTCGTCTGGTTAA<br>TGGCAGCCTGGCACTGATTTGGGATGATCTGCGTAGCCTGAGCCTTTTTAGCTATCATCGTCTG<br>CGTGATTTACTGCTGATTGTGACCCGTATCGTGGAACTGCTGGGCCGTCGT
**#4**	AATTGGCTGTGGTATATTCGTATTTTCATTATCATAGTGGGAAGCCTGATTGGCCTGCGTATTG<br>TGTTTGCGGTTCTGAGCCTGGTGAATCGGGTGCGTCAGGGTTATAGCCCGCTGAGCTTTCAGAC<br>CCATCTGCCTACCCCGCGTGGCGGAGAACGCGACCGTGATCGTAGCATTCGTTCTGTTAATGG<br>CAGCCTGGCATCTATTTGGGCGGCGCTGCGTGCGCTGAGCCTTTTTAGCTATCATCGTCTGCGT<br>GATTTACTGCTGATTGTGACCCGTATCGTGGAACTGCTGGGCCGTCGT
**#5**	AATTGGCTGTGGTATATTCGTATTTTCATTATCATAGTGGGAAGCCTGATTGGCCTGCGTATTG<br>TGTTTGCGGTTCTGAGCCTGGTGAATCGGGTGCGTCAGGGTTATAGCCCGCTGAGCTTTCAGAC<br>CCATCTGCCTACCCCGCGTGGCGGAGAACGCGACCGTGATCGTAGCATTCGTTCTGTTAATGG<br>CAGCCTGGCATCTATTTGGGCGGCGCTGCGTGCGCTGTGCCTTTTTAGCTATCATCGTCTGCGT<br>GATTTACTGCTGATTGTGACCCGTATCGTGGAACTGCTGGGCCGTCGT
**#6**	TGTCTGGAGCTGGACAAATGGTGTAGCCTGTGGAATTGGTTTGATATTACCAACTGGCTGTGGT<br>ATATTCGTATTTTCATCATTATTGTGGGCAGCCTGATTGGCCTGCGTATTGTGTTTGCGGTGCTG<br>AGCCTGGTGAATCGTGTGCGTCAG
**#7**	AATTGGCTGTGGTATATTCGTATTTTCATTATCATAGTGGGAAGCCTGATTGGCCTGCGTATTG<br>TGTTTGCGGTTCTGAGCCTGGTGAATCGGGTGCGTCAGGGTTATAGCCCGCTGAGCTTTCAGAC<br>CCATCTGCCTACCCCGCGTGGCGGAGAACGCGACCGTGATCGTAGCATTCGTCTGGTTAATGG<br>CAGCCTGGCACTGATTTGGGATGATCTGCGTAGCCTGAGCCTTTTTAGCTATCATCGTCTGCGT<br>GATTTACTGCTGATTGTGACCCGTATCGTGGAACTGCTGGGCCGTCGTGGCCGTCGTCATCACC<br>ATCACCATCAC
**#8**	GGCGGAGAACGCGACCGTGATCGTAGCATTCGTCTGGTTAATGGCAGCCTGGCACTGATTTGG<br>GATGATCTGCGTAGCCTGAGCCTTTTTAGCTATCATCGTCTGCGTGATTTACTGCTGATTGTGA<br>CCCGTATCGTGGAACTGCTGGGCCGTCGTCATCACCATCACCATCAC
	Primers
**#1**	GATCTGCGTAGCCTGAGCCTTTTTAGCTATCATCGTCTG
**#2**	CAGACGATGATAGCTAAAAAGGCTCAGGCTACGCAGATC
**#3**	CCGGATCGTCCGGAA GGCGGAGAACGCGA
**#4**	TCGCGTTCTCCGCCTTCCGGACGATCCGG
**#5**	CTTTCAGACCCATCTGCCTACCCCGCGTGGCGGAGAACGCGACCGTGATCGTAGCATTC
**#6**	GAATGCTACGATCACGGTCGCGTTCTCCGCCACGCGGGGTAGGCAGATGGGTCTGAAAG
**#7**	AACTGCTGGGCCGTCGTTGCTAAGGATCCGAATTCGAGC
**#8**	GCTCGAATTCGGATCCTTAGCAACGACGGCCCAGCAGTT

Supplementary Table 7 | NMR acquisition parameters.

Type of experiment	Spectral widths and chemical shift evolution times	# of scans	Inter-scan delay (s)	Duration of the experiment	Notes
Backbone assignment					
~ 0.7 mM (^{15}N, ^{13}C, 85% ^{2}H)-labeled TMD-CT^{LLP2}; magnetic field: 14.1 T					
2D ^{1}H-^{15}N TROSY HSQC	9600 Hz, 106.0 ms ($^{1}H^{N}$)<br>1600 Hz, 62.5 ms (^{15}N)	40	1.2	3 h	
3D TROSY HNCO	10800 Hz, 95.0 ms ($^{1}H^{N}$)<br>1600 Hz, 31.3 ms (^{15}N)<br>1700 Hz, 13.5 ms ($^{13}C'$)	32	1.2	2 d 10 h	
3D TROSY HNCA	10800 Hz, 95.0 ms ($^{1}H^{N}$)<br>1600 Hz, 31.3 ms (^{15}N)<br>5300 Hz, 5.3 ms ($^{13}C^{\alpha}$)	64	1.2	5 d 22 h	
~ 0.7 mM (^{15}N, ^{13}C, 85% ^{2}H)-labeled TMD-CT^{LLP2}; magnetic field: 17.6 T					
3D TROSY HN(CA)CO	10500 Hz, 97.0 ms ($^{1}H^{N}$)<br>1900 Hz, 30.0 ms (^{15}N)<br>1950 Hz, 16.4 ms ($^{13}C'$)	24	1.2	3 d	
3D TROSY HN(CO)CA	10500 Hz, 49.0 ms ($^{1}H^{N}$)<br>1800 Hz, 27.4 ms (^{15}N)<br>5000 Hz, 6.6 ms ($^{13}C^{\alpha}$)	20	1.2	2 d 3 h	
3D TROSY HNCACB	10500 Hz, 97.0 ms ($^{1}H^{N}$)<br>1900 Hz, 26.3 ms (^{15}N)<br>4700 Hz, 4.3 ms ($^{13}C^{\alpha/\beta}$)	32	1.3	2 d 8 h	
~ 0.8 mM (^{15}N, ^{2}H)-labeled TMD-CT^{LLP2}; magnetic field: 17.6 T					
3D ^{15}N-edited NOESY-TROSY-HSQC	10500 Hz, 95.0 ms ($^{1}H^{N}$)<br>1900 Hz, 28.9 ms (^{15}N)<br>2250 Hz, 20.0 ms (^{1}H)	20	1.2	3 d 15 h	τ_{mix} = 180 ms
Side-chain and NOE assignment					
~ 1.0 mM (^{15}N, ^{13}C)-labeled TMD-CT^{LLP2}; magnetic field: 18.8 T					
2D ^{1}H-^{15}N TROSY HSQC	11150 Hz, 92.0 ms ($^{1}H^{N}$)<br>1900 Hz, 134.7 ms (^{15}N)	32	1.2	6 h	
2D ^{1}H-^{13}C HSQC	11150 Hz, 46.0 ms ($^{1}H^{N}$)<br>4400 Hz, 27.3 ms (^{13}C)	32	1.2	3 h	
3D ^{15}N-edited NOESY-TROSY-HSQC	11150 Hz, 92.0 ms ($^{1}H^{N}$)<br>1900 Hz, 32.1 ms (^{15}N)<br>9600 Hz, 10.4 ms (^{1}H)	28	1.2	11 d 16 h	τ_{mix} = 80 ms
3D ^{13}C-edited NOESY	11150 Hz, 92.0 ms ($^{1}H^{N}$)<br>4400 Hz, 19.3 ms (^{13}C)<br>9600 Hz, 10.4 ms (^{1}H)	16	1.0	8 d 4 h	τ_{mix} = 150 ms

Supplementary Table 7 (continued)

Type of experiment	Spectral widths and chemical shift evolution times	# of scans	Inter-scan delay (s)	Duration of the experiment	Notes
~ 0.5 mM (^{15}N, ^{2}H)-labeled TMD-CT^{LLP2} (control sample); magnetic field: 21.1 T					
2D ^{1}H-^{15}N TROSY HSQC	12600 Hz, 81.0 ms ($^{1}H^{N}$)<br>1900 Hz, 73.1 ms (^{15}N)	8	1.3	1 h	
3D ^{15}N-edited NOESY-TROSY-HSQC	12600 Hz, 81.0 ms ($^{1}H^{N}$)<br>1900 Hz, 26.1 ms (^{15}N)<br>10800 Hz, 8.3 ms (^{1}H)	24	1.2	7 d 22 h	τ_{mix} = 200 ms
~ 0.5 mM (^{15}N, ^{2}H)-labeled TMD-CT^{LLP2} mixed with ~ 0.5 mM (^{13}C)-labeled TMD-CT^{LLP2} (~ 1:1); magnetic field: 21.1 T					
2D ^{1}H-^{15}N TROSY HSQC	12600 Hz, 81.0 ms ($^{1}H^{N}$)<br>1900 Hz, 73.1 ms (^{15}N)	8	1.3	1 h	
2D ^{1}H-^{13}C HSQC	12600 Hz, 40.5 ms ($^{1}H^{N}$)<br>4100 Hz, 27.8 ms (^{13}C)	8	1.3	1h	
3D ^{15}N-edited NOESY-TROSY-HSQC	12600 Hz, 81.0 ms ($^{1}H^{N}$)<br>1900 Hz, 26.1 ms (^{15}N)<br>10800 Hz, 8.3 ms (^{1}H)	24	1.2	7 d 22 h	τ_{mix} = 200 ms
~ 0.5 mM (^{15}N, ^{2}H)-labeled TMD-CT^{LLP2} mixed with ~ 0.5 mM (^{13}C)-labeled TMD-CT^{LLP2} (~ 1:1); magnetic field: 18.8 T					
2D ^{1}H-^{15}N TROSY HSQC	11150 Hz, 92.0 ms ($^{1}H^{N}$)<br>1900 Hz, 79.0 ms (^{15}N)	32	1.2	4 h	
2D ^{1}H-^{13}C HSQC	11150 Hz, 46.0 ms ($^{1}H^{N}$)<br>4400 Hz, 27.3 ms (^{13}C)	32	1.2	3 h	
3D J_{CH}-modulated NOESY	11150 Hz, 92.0 ms ($^{1}H^{N}$)<br>1900 Hz, 26.3 ms (^{15}N)<br>9600 Hz, 7.3 ms (^{1}H)	24	1.2	6 d 9 h (each spectrum)	τ_{mix} = 200 ms; two interleaved spectra were recorded (J_{CH} = 0 and 8 ms)
PPT					
~ 0.5 mM (^{15}N, 85% ^{2}H)-labeled TMD-CT^{LLP2}; magnetic field: 14.1 T					
2D ^{1}H-^{15}N TROSY HSQC	9600 Hz, 106.0 ms ($^{1}H^{N}$)<br>1600 Hz, 56.3 ms (^{15}N)	56	3.5	11 h (each spectrum)	[Gd-DOTA] = 0, 1.0, 2.0, 4.0, 6.0, 8.0, 10.0, 15.0, 20.0 mM
~ 0.9 mM (^{15}N, 85% ^{2}H)-labeled TMD-CT^{LLP2}; magnetic field: 14.1 T					
2D ^{1}H-^{15}N TROSY HSQC	9600 Hz, 106.0 ms ($^{1}H^{N}$)<br>1600 Hz, 56.3 ms (^{15}N)	32	3.5	6 h (each spectrum)	[16-DSA] = 0, 0.6, 1.2, 1.8, 2.4, 3.0, 3.6, 4.2 mM

Supplementary Table 7 (continued)

Type of experiment	Spectral widths and chemical shift evolution times	# of scans	Inter-scan delay (s)	Duration of the experiment	Notes
Inter-protomer PREs					
~ 0.3 mM (^{15}N, 85% ^{2}H)-labeled TMD-CT^{LLP2} (control sample); magnetic field: 14.1 T					
2D ^{1}H-^{15}N TROSY HSQC	9600 Hz, 106.0 ms ($^{1}H^{N}$)<br>1600 Hz, 93.8 ms (^{15}N)	56	3.5	17 h (each spectrum)	[Sodium ascorbate] = 0, 20.0 mM
~ 0.4 mM (^{15}N, 85% ^{2}H)-labeled TMD-CT^{LLP2} mixed with ~ 0.4 mM unlabeled TMD-CT^{LLP2} G738C (~ 1:1); magnetic field: 14.1 T					
2D ^{1}H-^{15}N TROSY HSQC	9600 Hz, 106.0 ms ($^{1}H^{N}$)<br>1600 Hz, 93.8 ms (^{15}N)	136	3.5	1 d 18 h (each spectrum)	[Sodium ascorbate] = 0, 20.0 mM
~ 0.4 mM (^{15}N, 85% ^{2}H)-labeled TMD-CT^{LLP2} mixed with ~ 0.4 mM unlabeled TMD-CT^{LLP2} S764C (~ 1:1); magnetic field: 14.1 T					
2D ^{1}H-^{15}N TROSY HSQC	9600 Hz, 106.0 ms ($^{1}H^{N}$)<br>1600 Hz, 93.8 ms (^{15}N)	196	3.5	1 d 6 h (each spectrum)	[Sodium ascorbate] = 0, 20.0 mM
~ 0.4 mM (^{15}N, 85% ^{2}H)-labeled TMD-CT^{LLP2} mixed with ~ 0.4 mM unlabeled TMD-CT^{LLP2} C789 (~ 1:1); magnetic field: 14.1 T					
2D ^{1}H-^{15}N TROSY HSQC	9600 Hz, 106.0 ms ($^{1}H^{N}$)<br>1600 Hz, 71.9 ms (^{15}N)	136	3.5	1 d 8 h (each spectrum)	[Sodium ascorbate] = 0, 20.0 mM
~ 0.5 mM (^{15}N, 85% ^{2}H)-labeled TMD-KS mixed with ~ 0.5 mM unlabeled CT^{LLP2} C789 (~ 1:1); magnetic field: 14.1 T					
2D ^{1}H-^{15}N TROSY HSQC	9600 Hz, 106.0 ms ($^{1}H^{N}$)<br>1600 Hz, 94.0 ms (^{15}N)	64	3.5	20 h (each spectrum)	[Sodium ascorbate] = 0, 20.0 mM
~ 0.15 mM (^{15}N, 85% ^{2}H)-labeled CT^{LLP2} mixed with ~ 0.35 mM unlabeled CT^{LLP2} C789 and ~ 0.5 mM unlabeled TMD-KS (~ 1:2:3); magnetic field: 14.1 T					
2D ^{1}H-^{15}N TROSY HSQC	9600 Hz, 106.0 ms ($^{1}H^{N}$)<br>1600 Hz, 94.0 ms (^{15}N)	64	3.5	20 h (each spectrum)	[Sodium ascorbate] = 0, 20.0 mM
~ 0.5 mM (15N, 85% 2H)-labeled CT2-tmd mixed with ~ 0.5 mM unlabeled CT2-tmd S764C (~ 1:1); magnetic field: 14.1 T					
2D ^{1}H-^{15}N TROSY HSQC	9600 Hz, 106.0 ms ($^{1}H^{N}$)<br>1600 Hz, 94.0 ms (^{15}N)	104	3.5	1 d 8 h (each spectrum)	[Sodium ascorbate] = 0, 20.0 mM
H-D exchange					
~ 0.8 mM (^{15}N, 85% ^{2}H)-labeled TMD-CT^{LLP2}; magnetic field: 14.1 T					
2D ^{1}H-^{15}N TROSY HSQC	9600 Hz, 106.0 ms ($^{1}H^{N}$)<br>1600 Hz, 75.0 ms (^{15}N)	32	1.2	3 h (each spectrum)	

Supplementary Table 7 (continued)

Type of experiment	Spectral widths and chemical shift evolution times	# of scans	Inter-scan delay (s)	Duration of the experiment	Notes
^{15}N backbone dynamics					
~ 0.5 mM (^{15}N, 85% ^{2}H)-labeled TMD-CT^{LLP2}; magnetic field: 14.1 T					
2D ^{1}H-^{15}N T_1 TROSY HSQC	9600 Hz, 106.0 ms ($^{1}H^{N}$) 1600 Hz, 75.0 ms (^{15}N)	40	1.5	6 h (average time of each spectrum)	Relaxation delays: 10, 50, 100, 200, 300, 600, 800, 1000 ms
2D ^{1}H-^{15}N T_2 TROSY HSQC	9600 Hz, 106.0 ms ($^{1}H^{N}$) 1600 Hz, 75.0 ms (^{15}N)	40	1.5	5 h (each spectrum)	Relaxation delays: 6.4, 10, 20, 30, 40, 50, 64, 80 ms
~ 0.6 mM (^{15}N, 85% ^{2}H)-labeled Unlocked MPER-TMD; magnetic field: 14.1 T					
2D ^{1}H-^{15}N T_1 TROSY HSQC	9600 Hz, 106.0 ms ($^{1}H^{N}$) 1500 Hz, 68.0 ms (^{15}N)	64	1.5	6 h (average time of each spectrum)	Relaxation delays: 10, 50, 100, 200, 300, 600, 800, 1000 ms
2D ^{1}H-^{15}N T_2 TROSY HSQC	9600 Hz, 106.0 ms ($^{1}H^{N}$) 1500 Hz, 68.0 ms (^{15}N)	80	1.5	9 h (each spectrum)	Relaxation delays: 6.4, 10, 20, 30, 40, 50, 64, 80 ms
~ 0.6 mM (^{15}N, 85% ^{2}H)-labeled Locked MPER-TMD; magnetic field: 14.1 T					
2D ^{1}H-^{15}N T_1 TROSY HSQC	9600 Hz, 106.0 ms ($^{1}H^{N}$) 1500 Hz, 68.0 ms (^{15}N)	64	1.5	6 h (average time of each spectrum)	Relaxation delays: 10, 50, 100, 200, 300, 600, 800, 1000 ms
2D ^{1}H-^{15}N T_2 TROSY HSQC	9600 Hz, 106.0 ms ($^{1}H^{N}$) 1500 Hz, 68.0 ms (^{15}N)	80	1.5	9 h (each spectrum)	Relaxation delays: 6.4, 10, 20, 30, 40, 50, 64, 80 ms

Supplementary Table 8 | Residue specific PRE_{amp} of TMD-CT^{LLP2} in q = 0.5 bicelles. The values were determined by fitting I/I_0 vs. [paramagnetic agent] to Eq. 1. PRE_{amp} of non-proline residues not shown were discarded because of signal overlap or poor data fitting.

Residue	PRE_{amp} (Gd-DOTA)	PRE_{amp} (16-DSA)	Residue	PRE_{amp} (Gd-DOTA)	PRE_{amp} (16-DSA)
679	0.914±0.036	0.871±0.011	716	1.011±0.019	0.883±0.023
680	0.946±0.063	-	717	0.987±0.026	0.795±0.012
681	0.943±0.052	0.9015±0.0080	718	0.934±0.025	0.772±0.022
682	-	0.912±0.024	719	0.964±0.043	-
684	0.857±0.054	0.930±0.012	721	0.992±0.015	0.733±0.017
685	-	0.966±0.020	723	0.951±0.009	-
686	0.763±0.057	0.9869±0.0093	725	0.974±0.020	-0.208±0.027 (*)
688	0.722±0.029	0.974±0.023	737	0.961±0.040	-
689	-	0.987±0.013	738	0.999±0.037	-2.66±0.44 (*)
690	0.654±0.058	0.9973±0.0082	739	0.977±0.023	-0.636±0.021 (*)
693	0.653±0.063	0.980±0.021	744	0.959±0.018	0.380±0.016 (*)
694	0.652±0.046	0.9930±0.0047	745	0.989±0.024	0.793±0.031
695	0.662±0.041	0.990±0.013	746	-	0.856±0.018
696	0.661±0.045	0.970±0.019	747	0.969±0.023	0.871±0.024
698	0.655±0.057	0.975±0.022	748	0.942±0.035	0.929±0.021
699	0.683±0.025	0.981±0.013	750	0.946±0.053	0.957±0.014
700	0.709±0.027	0.966±0.020	751	0.949±0.021	0.907±0.028
701	-	0.987±0.013	753	0.910±0.033	0.960±0.015
702	0.771±0.061	0.981±0.012	754	0.907±0.028	0.921±0.022
703	0.864±0.043	0.950±0.022	755	0.933±0.023	0.946±0.023
704	0.879±0.057	0.944±0.021	756	0.871±0.023	0.955±0.020
705	0.907±0.066	0.945±0.019	758	0.870±0.036	0.925±0.028
706	0.908±0.055	0.904±0.015	759	-	0.923±0.020
707	-	0.891±0.010	762	0.909±0.043	0.949±0.022
708	0.920±0.055	0.889±0.016	763	0.932±0.054	-
709	-	0.895±0.032	764	0.987±0.044	0.950±0.019
710	0.983±0.024	0.812±0.011	770	0.981±0.035	0.963±0.011
711	1.008±0.019	0.844±0.016	771	0.892±0.075	0.918±0.023
712	0.950±0.014	0.8611±0.0099	772	0.930±0.079	0.938±0.026
713	0.981±0.014	0.858±0.015	773	0.946±0.055	0.865±0.022
715	0.977±0.018	0.909±0.017	774	0.891±0.097	0.922±0.020

Supplementary Table 8 (continued)

775	0.869±0.060	0.967±0.020	782	0.923±0.088	0.931±0.021
776	0.901±0.034	0.9264±0.0092	784	0.953±0.075	0.835±0.015
777	0.922±0.090	0.908±0.017	785	0.925±0.065	0.8591±0.0079
778	0.899±0.088	0.959±0.024	786	0.960±0.071	0.877±0.020
779	0.92±0.10	0.926±0.027	787	1.100±0.057	0.820±0.011
780	0.938±0.074	0.870±0.020	788	1.033±0.044	0.754±0.022

(*) These PRE_{amp} were not used in the PPT analysis since their values were dramatically dominated by the fast timescale of the highly flexible loop.

Supplementary Table 9 | Residue-specific k_{ex} of the TMD-CT^{LLP2} in bicelles. k_{ex} of non-proline residues not shown were discarded because of signal overlap or poor data fitting. '*' indicates residues with k_{ex} too fast to be measured. The colored boxes represent the four different exchange regimes defined as: very fast (τ_{ex} < 1 hour) (red), fast (1 hour ≤ τ_{ex} < 3 hours) (orange), slow (3 hours ≤ τ_{ex} < 1 day) (yellow), and very slow (τ_{ex} ≥ 1 day) (white).

Residue (H^N)	k_{ex} (Hz)	τ_{ex}
679	*	*
681	*	*
684	$(2.3±1.1)·10^{-4}$	1.2±0.6 hours
685	$(6.1±1.4)·10^{-5}$	4.6±1.1 hours
686	$(6.4±1.6)·10^{-6}$	1.8±0.4 days
687	$(1.6±0.4)·10^{-5}$	17.1±4.0 hours
688	$(3.7±1.5)·10^{-6}$	3.1±1.2 days
689	$(2.4±0.9)·10^{-6}$	4.8±1.7 days
690	$(5.2±0.5)·10^{-5}$	5.3±0.6 hours
693	$(1.0±0.3)·10^{-4}$	2.7±0.8 hours
694	$(9.9±6.1)·10^{-5}$	2.8±1.8 hours
695	*	*
698	$(1.7±0.2)·10^{-4}$	1.6±0.2 hours
699	$(1.0±0.7)·10^{-4}$	2.8±1.9 hours
700	*	*
701	$(1.5±0.2)·10^{-4}$	1.9±0.2 hours
702	$(2.2±0.6)·10^{-4}$	1.3±0.3 hours
703	*	*
704	$(2.3±0.6)·10^{-5}$	12.3±3.4 hours
705	$(2.6±1.6)·10^{-5}$	10.6±6.5 hours
706	$(1.2±0.5)·10^{-4}$	2.3±1.0 hours
707	*	*
708	*	*
710	*	*
711	*	*
712	*	*
713	*	*
715	*	*
716	*	*
717	*	*
718	*	*
719	*	*
721	*	*
723	*	*
725	*	*
737	$(2.4±1.2)·10^{-4}$	1.2±0.6 hours
738	*	*
739	*	*
741	*	*
742	*	*
744	*	*
745	$(1.9±0.1)·10^{-5}$	14.3±7.8 hours
747	*	*
748	$(7.3±3.8)·10^{-5}$	3.8±2.0 hours
750	*	*
751	$(8.3±3.9)·10^{-5}$	3.4±1.6 hours
753	$(1.0±0.6)·10^{-4}$	2.7±1.6 hours
754	*	*
755	$(2.9±1.5)·10^{-5}$	9.7±5.1 hours
756	$(1.5±1.1)·10^{-4}$	1.8±1.3 hours
758	$(1.1±0.4)·10^{-4}$	2.5±0.9 hours
760	$(3.9±0.5)·10^{-4}$	7.2±1.0 hours
762	*	*
763	*	*
764	$(9.6±4.1)·10^{-5}$	2.9±1.2 hours
770	*	*
772	$(2.1±1.1)·10^{-4}$	1.3±0.7 hours
773	$(4.0±1.4)·10^{-5}$	7.0±2.4 hours
774	$(3.1±2.0)·10^{-5}$	8.9±5.6 hours
775	$(1.3±0.5)·10^{-4}$	2.1±0.8 hours
777	$(2.1±0.9)·10^{-5}$	13.4±5.8 hours
778	$(2.3±0.5)·10^{-4}$	1.2±0.3 hours
779	*	*
780	*	*
781	$(1.7±0.7)·10^{-4}$	1.6±0.7 hours
782	$(1.1±0.1)·10^{-4}$	2.6±0.4 hours
783	*	*
784	*	*
785	*	*
786	*	*
787	*	*
788	*	*

Supplementary Table 10 | Plane restraints used for the TMD-CT^{LLP2} and MPER-TMD-CT^{LLP2} structure calculation. $r_Z = 0$ corresponds to the bicelle center. Plane restraints for the MPER and CT are shown in light blue and green, respectively; those for the TMD structure (internal calibration) are shown in red.

Residue	TMD-CT^{LLP2} r_Z (Å)	MPER-TMD-CT^{LLP2} r_Z (Å)	Residue	TMD-CT^{LLP2} r_Z (Å)	MPER-TMD-CT^{LLP2} r_Z (Å)
678	-	17.8±1.0	709	-22.8±1.0	-22.8±1.0
679	24.1±1.0	18.4±1.0	710	-23.8±1.0	-23.8±1.0
680	23.5±1.0	17.5±1.0	712	-23.3±1.0	-23.3±1.0
681	21.5±1.0	18.1±1.0	713	-23.5±1.0	-23.5±1.0
682	19.7±1.0	17.8±1.0	715	-20.3±1.0	-20.3±1.0
683	19.1±1.0	16.5±1.0	716	-22.0±1.0	-22.0±1.0
684	17.7±1.0	16.7±1.0	746	-23.6±1.0	-23.6±1.0
685	15.5±1.0	15.3±1.0	747	-22.7±1.0	-22.7±1.0
686	14.0±1.0	13.5±1.0	748	-18.9±1.0	-18.9±1.0
687	13.0±1.0	12.6±1.0	751	-20.4±1.0	-20.4±1.0
688	11.2±1.0	-	753	-18.7±1.0	-18.7±1.0
689	9.3±1.0	-	754	-19.0±1.0	-19.0±1.0
690	8.5±1.0	-	755	-17.5±1.0	-17.5±1.0
691	6.7±1.0	-	756	-16.8±1.0	-16.8±1.0
692	4.3±1.0	-	758	-18.0±1.0	-18.0±1.0
693	3.0±1.0	-	759	-19.4±1.0	-19.4±1.0
694	1.8±1.0	-	762	-18.0±1.0	-18.0±1.0
695	-0.4±1.0	-0.4±1.0	771	-18.8±1.0	-18.8±1.0
696	-2.4±1.0	-2.4±1.0	772	-19.2±1.0	-19.2±1.0
697	-4.3±1.0	-4.3±1.0	773	-23.1±1.0	-23.1±1.0
698	-6.6±1.0	-6.6±1.0	774	-18.6±1.0	-18.6±1.0
699	-8.4±1.0	-8.4±1.0	775	-16.8±1.0	-16.8±1.0
700	-10.0±1.0	-10.0±1.0	776	-18.7±1.0	-18.7±1.0
701	-11.7±1.0	-11.7±1.0	777	-20.0±1.0	-20.0±1.0
702	-13.1±1.0	-13.1±1.0	778	-18.1±1.0	-18.1±1.0
703	-15.2±1.0	-15.2±1.0	779	-19.2±1.0	-19.2±1.0
704	-17.6±1.0	-17.6±1.0	780	-22.7±1.0	-22.7±1.0
705	-18.2±1.0	-18.2±1.0	782	-19.2±1.0	-19.2±1.0
706	-18.9±1.0	-18.9±1.0	785	-23.4±1.0	-23.4±1.0
707	-21.3±1.0	-21.3±1.0	786	-22.3±1.0	-22.3±1.0
708	-23.2±1.0	-23.2±1.0			

Supplementary Table 11 | TMD-CT^{LLP2} NMR structure calculation and refinement statistics.

**NMR distance, dihedral and plane restraints** [a]	**TMD (677-710)**	**TMD-CT^{LLP2} (711-788)**
Distance restraints from NOE [b]	510	585
Short-range intramolecular ($\|i\text{-}j\| \leq 4$)	459	498
Long-range intramolecular ($\|i\text{-}j\| \geq 5$)	0	24
Intermolecular	51	63
Total plane restraints [c]	96	84
Total dihedral angle restraints [d]	480	
Φ (TALOS)	240	
ψ (TALOS)	240	
**Structure statistics** [e]		
Violations (mean ± s.d.)		
Distance restraints (Å)	0.131±0.006	
Plane restraints (Å)	0.063±0.026	
Dihedral angle restraints (°)	0.686±0.065	
Deviations from idealized geometry		
Bond lengths (Å)	0.008±0.000	
Bond angles (°)	0.917±0.015	
Impropers (°)	0.621±0.022	
Average pairwise r.m.s. deviation (Å) [f]		
Heavy	1.763	
Backbone	1.296	

[a] The numbers of restraints are summed over all three subunits.

[b] For residues 711-788, all NOE restraints were obtained in the current study. For residues 679-710, the NOE restraints were taken from our earlier NMR study of the TMD[3].

[c] For residues 711-788, plane restraints were derived using PRE_{amp} values obtained in the current study. For residues 679-710, plane restraints were directly taken from our earlier NMR study of the TMD (internal calibration)[4].

[d] Backbone φ and ψ restraints and their respective uncertainties were obtained from the "GOOD" dihedrals generated by *TALOS+* based on the backbone chemical shift values.

[e] Statistics are calculated and averaged over an ensemble of the 15 lowest energy structures out of 150 calculated structures.

[f] The precision of the atomic coordinates is defined as the average r.m.s. difference between the 15 final structures and their mean coordinates (flexible residues 711-740 have been omitted from the comparison).

Supplementary Table 12 | MPER-TMD-CT^{LLP2} NMR model calculation and refinement statistics.

**NMR distance, dihedral and plane restraints** [a]	**MPER-TMD (660-693)**	**TMD (694-710)**	**TMD-CT^{LLP2} (711-788)**
Distance restraints from NOE [b]	333	429	585
Short-range intramolecular (|*i*-*j*|≤4)	303	387	498
Long-range intramolecular (|*i*-*j*|≥5)	6	0	24
Intermolecular	24	42	63
Total plane restraints [c]	30	48	84
Total dihedral angle restraints [d]		540	
Φ (TALOS)		270	
ψ (TALOS)		270	
**Structure statistics** [e]			
Violations (mean ± s.d.)			
Distance restraints (Å)		0.118±0.003	
Plane restraints (Å)		0.074±0.020	
Dihedral angle restraints (°)		0.728±0.038	
Deviations from idealized geometry			
Bond lengths (Å)		0.008±0.000	
Bond angles (°)		0.886±0.018	
Impropers (°)		0.606±0.024	
Average pairwise r.m.s. deviation (Å) [f]			
Heavy		1.240	
Backbone		0.941	

[a] The numbers of restraints are summed over all three subunits.

[b] For residues 711-788, all NOE restraints were obtained in the current study. For the MPER and first half of the TMD (residues 660-693), the NOE restraints were taken from our earlier NMR study of the MPER-TMD[5]; for the second half of the TMD (residues 694-710), the NOE restraints were taken from our earlier NMR study of the TMD[3].

[c] For the residues 711-788, plane restraints were derived using the PRE_{amp} values obtained in the current study. For the MPER and first half of the TMD (residues 660-693), plane restraints were derived using the PRE_{amp} values taken from our earlier NMR study of the MPER-TMD[5]; for the second half of the TMD (residues 694-710), plane restraints were directly taken from our earlier NMR study of the TMD (internal calibration)[4].

[d] Backbone φ and ψ restraints and their respective uncertainties were obtained from the "GOOD" dihedrals generated by *TALOS+* based on the backbone chemical shift values. For residues 694-788, all dihedral angle restraints were obtained in the current study; for residues 660-693, all dihedral angle restraints were taken from our earlier NMR study of the MPER-TMD[5].

[e] Statistics are calculated and averaged over an ensemble of the 15 lowest energy structures out of 150 calculated structures.

[f] The precision of the atomic coordinates is defined as the average r.m.s. difference between the 15 final structures and their mean coordinates (flexible residues 711-740 have been omitted from the comparison).

Supplementary Table 13 | DNA sequence of the synthetic genes used to generate the expression constructs of antibodies PG9, PG16, and PGT145.

Synthetic genes	
**PG9**	See reference [11].
**PG16**	See reference [11].
**PGT145**	*Heavy chain*:<br>GGGGACAAGTTTGTACAAAAAAGCAGGCTGAGCTCTCTAGAGAATTCGCCACCATGG<br>GCTGGTCCTGCATCATCCTGTTTCTGGTGGCCACAGCCACCGGCGTGCACAGCCAGGC<br>CTCTACCATGGACTGGATCTGGCGGATTCTGTTCCTGGTGGCTGCCGCCACAAGCGCC<br>CATTCTCAGGTGCAGCTGGTGCAGTCTGGCGCCGAAGTGAAGAAACCCGGCAGCAGC<br>GTGAAGGTGTCCTGCAAGGCCAGCGGCAACAGCTTCAGCAACCACGACGTGCACTGG<br>GTGCGCCAGGCTACAGGACAGGGCCTGGAATGGATGGGCTGGATGAGCCACGAGGGC<br>GACAAGACAGGACTGGCCCAGAAATTCCAGGGCAGAGTGACCATCACCCGGGACAGC<br>GGAGCCAGCACCGTGTACATGGAACTGAGAGGCCTGACCGCCGACGACACCGCCATC<br>TACTACTGTCTGACCGGCAGCAAGCACCGGCTGCGGGACTACTTCCTGTACAACGAGT<br>ACGGCCCCAACTACGAGGAATGGGGCGACTACCTGGCCACCCTGGATGTGTGGGGAC<br>ACGGAACAGCCGTGACCGTGTCTAGCGCCAGCACAAAGGGCCCCAGCGTGTTCCCTCT<br>GGCCCCTAGCAGCAAGAGCACATCTGGCGGCACAGCCGCCCTGGGCTGCCTCGTGAA<br>GGATTACTTCCCCGAACCCGTGACAGTGTCCTGGAACTCTGGCGCCCTGACCAGCGGA<br>GTGCACACCTTTCCAGCCGTGCTGCAGAGCAGCGGCCTGTACTCTCTGAGCAGCGTCG<br>TGACTGTGCCCAGCAGCAGCCTGGGAACCCAGACCTACATCTGCAACGTGAACCACA<br>AGCCCAGCAACACCAAGGTGGACAAGAAGGTGGAACCCAAGAGCTGCGACTGATGAG<br>GATCCGCTAGCGGTACCACCCAGCTTTCTTGTACAAAGTGGTCCCC
	Light chain:<br>GGGGACAAGTTTGTACAAAAAAGCAGGCTGAGCTCTCTAGAGAATTCGCCACCATGG<br>GCTGGTCCTGCATCATCCTGTTTCTGGTGGCCACAGCCACCGGCGTGCACAGCATGAG<br>ACTGCCTGCTCAGCTGCTGGGCCTGCTGATGCTGTGGGTGTCAGGATCTGGCGCCGAG<br>GTCGTGATTACCCAGAGCCCCCTGTTCCTGCCTGTGACACCTGGCGAAGCCGCCAGCC<br>TGAGCTGCAAGTGTAGCCACAGCCTGCAGCACTCCACCGGCGCCAATTACCTGGCCTG<br>GTATCTGCAGCGGCCTGGCCAGACACCCAGACTGCTGATTCACCTGGCCACCCACAGA<br>GCCTCTGGCGTGCCCGATAGATTTTCCGGCTCTGGCAGCGGCACCGACTTCACCCTGA<br>AGATCAGCCGGGTGGAAAGCGACGACGTGGGCACCTACTACTGTATGCAGGGCCTGC<br>ACAGCCCCTGGACCTTTGGCCAGGGCACCAAGGTGGAAATCAAGCGGACAGTGGCCG<br>CTCCCAGCGTGTTCATCTTCCCACCTAGCGACGAGCAGCTGAAGTCCGGCACAGCCTC<br>TGTCGTGTGCCTGCTGAACAACTTCTACCCCCGCGAGGCCAAGGTGCAGTGGAAGGTG<br>GACAATGCCCTGCAGAGCGGCAACAGCCAGGAAAGCGTGACCGAGCAGGACAGCAA<br>GGACTCCACCTACAGCCTGTCCAGCACCCTGACCCTGAGCAAGGCCGACTACGAGAA<br>GCACAAGGTGTACGCCTGCGAAGTGACCCACCAGGGCCTGTCTAGCCCCGTGACCAA<br>GAGCTTCAACCGGGGCGAGTGCTGATGAGGATCCGCTAGCGGTACCACCCAGCTTTCT<br>TGTACAAAGTGGTCCCC

Supplementary References

1 Chen, W. *et al.* The Unusual Transmembrane Partition of the Hexameric Channel of the Hepatitis C Virus. *Structure* **26**, 627-634 e624 (2018).

2 Shen, Y. & Bax, A. Identification of helix capping and b-turn motifs from NMR chemical shifts. *Journal of biomolecular NMR* **52**, 211-232 (2012).

3 Dev, J. *et al.* Structural basis for membrane anchoring of HIV-1 envelope spike. *Science* **353**, 172-175 (2016).

4 Piai, A., Dev, J., Fu, Q. & Chou, J. J. Stability and Water Accessibility of the Trimeric Membrane Anchors of the HIV-1 Envelope Spikes. *Journal of the American Chemical Society* **139**, 18432-18435 (2017).

5 Fu, Q. *et al.* Structure of the membrane proximal external region of HIV-1 envelope glycoprotein. *Proceedings of the National Academy of Sciences of the United States of America* **115**, E8892-E8899 (2018).

6 Gristick, H. B. *et al.* Natively glycosylated HIV-1 Env structure reveals new mode for antibody recognition of the CD4-binding site. *Nature structural & molecular biology* **23**, 906-915 (2016).

7 Liu, J., Bartesaghi, A., Borgnia, M. J., Sapiro, G. & Subramaniam, S. Molecular architecture of native HIV-1 gp120 trimers. *Nature* **455**, 109-113 (2008).

8 Munoz, V. & Serrano, L. Elucidating the folding problem of helical peptides using empirical parameters. *Nat Struct Biol* **1**, 399-409 (1994).

9 Raghava, G. P. S. Protein secondary structure prediction using nearest neighbor and neural network approach. (2000).

10 Jones, D. T. Protein secondary structure prediction based on position-specific scoring matrices. *J Mol Biol* **292**, 195-202 (1999).

11 Walker, L. M. *et al.* Broad and potent neutralizing antibodies from an African donor reveal a new HIV-1 vaccine target. *Science* **326**, 285-289 (2009).
