# Supplementary material for: Structural basis of transmembrane coupling of the HIV-1 envelope glycoprotein
Source: Nat Commun. 2020 May 8;11:2317. doi: 10.1038/s41467-020-16165-0 (PMC7210310; doi:10.1038/s41467-020-16165-0)
Supplement: Supplementary file 3 — Reporting summary [file 41467_2020_16165_MOESM3_ESM.pdf]

nature research

Corresponding author(s): James J. Chou

Last updated by author(s): Apr 8, 2020

# Reporting Summary

Nature Research wishes to improve the reproducibility of the work that we publish. This form provides structure for consistency and transparency in reporting. For further information on Nature Research policies, see Authors & Referees and the Editorial Policy Checklist .

## Statistics

For all statistical analyses, confirm that the following items are present in the figure legend, table legend, main text, or Methods section.

| n/a | Confirmed | |
|---|---|---|
| ☐ | ☒ | The exact sample size (*n*) for each experimental group/condition, given as a discrete number and unit of measurement |
| ☐ | ☒ | A statement on whether measurements were taken from distinct samples or whether the same sample was measured repeatedly |
| ☒ | ☐ | The statistical test(s) used AND whether they are one- or two-sided<br>*Only common tests should be described solely by name; describe more complex techniques in the Methods section.* |
| ☒ | ☐ | A description of all covariates tested |
| ☒ | ☐ | A description of any assumptions or corrections, such as tests of normality and adjustment for multiple comparisons |
| ☐ | ☒ | A full description of the statistical parameters including central tendency (e.g. means) or other basic estimates (e.g. regression coefficient) AND variation (e.g. standard deviation) or associated estimates of uncertainty (e.g. confidence intervals) |
| ☒ | ☐ | For null hypothesis testing, the test statistic (e.g. *F*, *t*, *r*) with confidence intervals, effect sizes, degrees of freedom and *P* value noted<br>*Give P values as exact values whenever suitable.* |
| ☒ | ☐ | For Bayesian analysis, information on the choice of priors and Markov chain Monte Carlo settings |
| ☒ | ☐ | For hierarchical and complex designs, identification of the appropriate level for tests and full reporting of outcomes |
| ☒ | ☐ | Estimates of effect sizes (e.g. Cohen's *d*, Pearson's *r*), indicating how they were calculated |

*Our web collection on statistics for biologists contains articles on many of the points above.*

## Software and code

Policy information about availability of computer code

| | |
|---|---|
| Data collection | Bruker Biospin software TopSpin for NMR data collection. |
| Data analysis | NMR data processing and analysis: nmrPipe, SPARKY, XEASY, Origin |

For manuscripts utilizing custom algorithms or software that are central to the research but not yet described in published literature, software must be made available to editors/reviewers. We strongly encourage code deposition in a community repository (e.g. GitHub). See the Nature Research guidelines for submitting code & software for further information.

## Data

Policy information about availability of data

All manuscripts must include a data availability statement. This statement should provide the following information, where applicable:

- Accession codes, unique identifiers, or web links for publicly available datasets
- A list of figures that have associated raw data
- A description of any restrictions on data availability

Protein structure coordinates have been deposited to PDB, and NMR chemical shift values have been deposited to BMRB. Other data that support the findings of this study are available from the corresponding author upon reasonable request.

# Field-specific reporting

Please select the one below that is the best fit for your research. If you are not sure, read the appropriate sections before making your selection.

☒ Life sciences ☐ Behavioural & social sciences ☐ Ecological, evolutionary & environmental sciences

For a reference copy of the document with all sections, see nature.com/documents/nr-reporting-summary-flat.pdf

# Life sciences study design

All studies must disclose on these points even when the disclosure is negative.

| | |
|---|---|
| Sample size | For the functional mutagenesis studies, results of all 28 mutants have been included, none have been excluded. For structure determination, sample size is not applicable. |
| Data exclusions | N/A |
| Replication | The functional experiments were carried out in triplicate and repeated at least twice. |
| Randomization | N/A |
| Blinding | N/A |

# Reporting for specific materials, systems and methods

We require information from authors about some types of materials, experimental systems and methods used in many studies. Here, indicate whether each material, system or method listed is relevant to your study. If you are not sure if a list item applies to your research, read the appropriate section before selecting a response.

## Materials & experimental systems

| n/a | Involved in the study | |
|---|---|---|
| ☐ | ☒ | Antibodies |
| ☐ | ☒ | Eukaryotic cell lines |
| ☒ | ☐ | Palaeontology |
| ☒ | ☐ | Animals and other organisms |
| ☒ | ☐ | Human research participants |
| ☒ | ☐ | Clinical data |

## Methods

| n/a | Involved in the study | |
|---|---|---|
| ☒ | ☐ | ChIP-seq |
| ☐ | ☒ | Flow cytometry |
| ☒ | ☐ | MRI-based neuroimaging |

## Antibodies

| | |
|---|---|
| Antibodies used | We have generated the expression construct of antibody using synthetic genes made by GeneArt Gene Synthesis (Life Technologies). The plasmid was transfected to HEK 293T cells for expression, followed by purification using CaptureSelect beads provided by ThermoScientific. |
| Validation | Antibodies used including IgG of 2G12, b6, 3791, 17b, PG9, PG16, PGT145 and VRC01 were validated by binding to HIV-1 Env proteins, as well as by a luciferase-based neutraliztion assay with Env pseudoviruses in TZM.bl cells. |

## Eukaryotic cell lines

Policy information about cell lines

| | |
|---|---|
| Cell line source(s) | HEK 293T cells were purchased from ATCC; Expi293F from Thermo Fisher Scientific. |
| Authentication | Each cell line was authenticated for protein expression by western blot and/or flow cytometry, and other functional assays, such as a cell-cell fusion assay. |
| Mycoplasma contamination | Mycoplasma contamination is routinely tested for our cell culture and no contaminated cells were ever used for our studies. |
| Commonly misidentified lines (See ICLAC register) | None. |

# Flow Cytometry

## Plots

Confirm that:

- [ ] The axis labels state the marker and fluorochrome used (e.g. CD4-FITC).
- [x] The axis scales are clearly visible. Include numbers along axes only for bottom left plot of group (a 'group' is an analysis of identical markers).
- [ ] All plots are contour plots with outliers or pseudocolor plots.
- [ ] A numerical value for number of cells or percentage (with statistics) is provided.

## Methodology

| | |
|---|---|
| Sample preparation | 293T cells were transiently transfected with 2 µg of the 92UG037.8 gp160 expression construct or its CT mutants in 6 wells plates. Env-expressing cells were detached from plates using PBS, and washed with ice-cold PBS containing 1% BSA. 106 cells were incubated for 30~40 minutes on ice with either VRC01 Fab, 2G12 Fab, or PG16 IgG at concentrations of 10 µg/ml in PBS containing 1% BSA. The cells were then washed twice with PBS containing 1% BSA and stained with R-Phycoerythrin AffiniPure F(ab')2 fragment goat anti-human IgG, F(ab')2 Fragment specific secondary antibody (Jackson ImmunoResearch laboratories, West Grove, PA) at 5 µg/ml. All the fluorescently labeled cells were washed twice with PBS containing 1% BSA and analyzed immediately using a BD LSRII instrument and program FACSDIVA (BD Biosciences, San Jose, CA). All data were analyzed by FlowJo (FlowJo, LLC, Ashland, OR). |
| Instrument | BD FACScanto II |
| Software | FACSDIVA and Flowjo |
| Cell population abundance | N/A |
| Gating strategy | Only gating used during analysis is to separate live and single cell population. |

- [x] Tick this box to confirm that a figure exemplifying the gating strategy is provided in the Supplementary Information.
